# Low initial metabolite production enhances stability in syntrophic bacterial consortia

**DOI:** 10.1038/s42003-026-10187-y

**Published:** 2026-05-06

**Authors:** Nan Ye, Derek W. Dunn, Zhichun Yang, Beibei Hou, Huan Wang, Jianxiao Song, Rui-Wu Wang

**Affiliations:** 1 Center for Materials Synthetic Biology, Shenzhen Institute of Synthetic Biology, Shenzhen Institute of Advanced Technology, Chinese Academy of Sciences, Shenzhen, China; 2 State Key Laboratory of Quantitative Synthetic Biology, Shenzhen Institute of Synthetic Biology, Shenzhen Institutes of Advanced Technology, Chinese Academy of Sciences, Shenzhen, China; 3 School of Life Science and Technology, Northwestern Polytechnical University, Xi’an, China; 4 College of Life Sciences, Northwest University, Xi’an, China; 5 College of Life Sciences, Zhejiang University, Hangzhou, China

**Keywords:** Experimental evolution, Microbial ecology, Microbial ecology, Evolutionary ecology

## Abstract

Syntrophic interactions based on reciprocal metabolite exchange are widespread in microbial communities, yet the factors determining their stability remain unclear. Using synthetic *Escherichia coli* consortia composed of lysine and arginine auxotrophs, we show that lower initial metabolite production promotes, rather than limits, syntrophic stability. During serial propagation, replicate cocultures diverged sharply: a minority maintained sustained growth, whereas most became extinct. This divergence was associated with phenotypic differences in metabolite production among founding isolates. Consortia founded by low-producing strains recovered reliably after dilution and were more resistant to invasion by non-producing mutants. By contrast, high-producing founder generated diminishing returns for consortium growth, and increased extracellular metabolite availability that favored exploitation by non-producer. Although we detected no consistent coding-region variations between high- and low-producing isolates, expression differences suggest that outside coding regions may influence these production traits. These results identify constrained initial metabolite production as a key determinant of syntrophic stability.

## Abstract (web summary)

Low initial metabolite production stabilizes syntrophic *E. coli* consortia by constraining metabolite availability, promoting localized exchange, and resisting invasion by non-producers

## Introduction

Stability is a defining feature of ecological communities and determines whether they persist under environmental change and evolutionary pressure^1–3^. In microbial systems, stability is often discussed in two related ways. Ecological stability describes whether a community maintains its composition and function after disturbance, whereas evolutionary stability concerns whether within and among-species traits are maintained by selection over time^4,5^. Both ecological and evolutionary stability depend on a network of interactions among community members and on how those interactions respond to perturbation^1,6,7^. Syntrophic interactions through reciprocal metabolite exchange are widespread in both natural and engineered microbial communities^8^. Such interactions can broaden the effective metabolic capabilities of individual strains, improve resource use under nutrient limitation, and buffer communities against environmental variability^9,10^. For these reasons, syntrophy (also termed “cross-feeding”) has become central to microbial ecology and is often used as a design principle in synthetic communities^11,12^.

Syntrophic interactions frequently arise through auxotroph, in which organisms lose biosynthetic capabilities and depend on metabolites produced by neighboring cells^13,14^. Comparative genomics suggests that auxotroph is frequent across bacterial lineages, particularly for amino acids^15,16^. Biosynthetic gene loss can be favoured when it reduces biosynthetic costs if the required metabolites still remain accessible from the environment or surrounding cells^10,15^. This framework has been influential in explaining the prevalence of metabolic dependencies in microbial communities and has motivated the construction of auxotroph-based consortia in both ecological and biotechnological contexts^10,17,18^.

However, stable cross-feeding between different microbial strains is difficult to obtain in the laboratory^19^. Large screens of auxotrophic cocultures show that even known complementary pairs of strains often fail to coexist through repeated dilution-regrowth cycles^11^. For example, in yeast unless one partner is engineered to overproduce the exchanged metabolite, initial synthetic mutually beneficial interacting strains in minimal medium frequently collapse, a process characterized by population decline and extinction over serial transfers^20^. These results have supported a widely held notion that increasing metabolite supply enhances the mutual exchange of beneficial metabolites and thus promote stable mutualism between strains^21^.

More recent work indicates that cooperation between microbial strains can also be strengthened without one strain being engineered to overproduce metabolite in order to benefit another strain^11,22^. In amino-acid auxotrophic *E. coli*, serial passaging can select for reciprocal fitness feedbacks that increase coculture performance^22^. Similarly, a few auxotrophic yeast pairs, without overexpression modifications, can restore stable strain ratios via metabolic adaptation even when their initial proportions deviate from optimal values^11^. These examples show that stable cross-feeding can be achieved through the inherent capacity for synergistic regulation of metabolic flux of different microbial strains, rather than relying solely on the mandatory control imposed by genetic circuits^11^. These observations raise the question: what factors enable stable metabolite cross-feeding between different microbial strains?

**Fig. 1:**
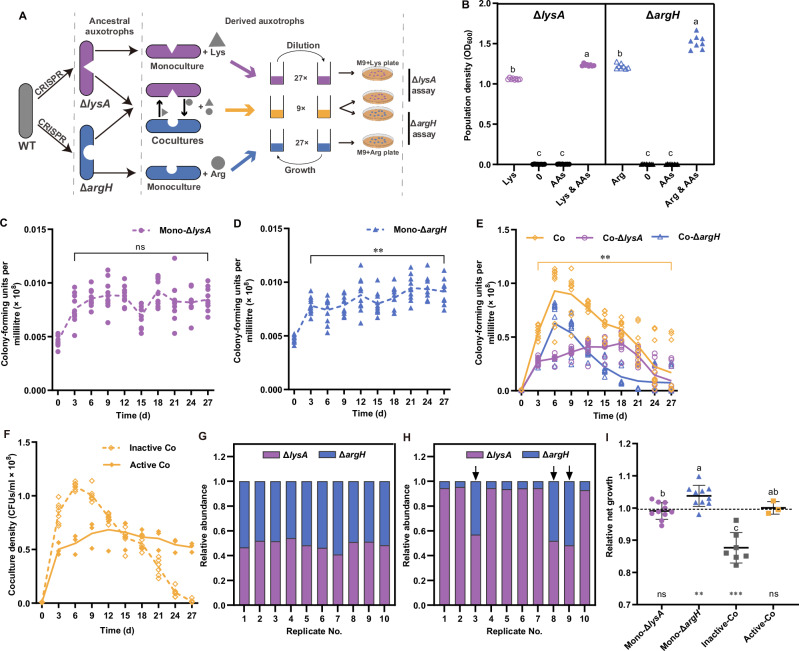
Growth dynamics and evolutionary outcomes of auxotrophic monocultures and cocultures. **A** Experimental design of the laboratory evolution experiment. Lysine- and arginine-auxotrophic *E. coli* (Δ*lysA* and Δ*argH*) were generated from a prototrophic *E. coli* (WT) and propagated either as monocultures supplemented with the lysine (+Lys) or arginine (+Arg) or as cocultures supplemented with both amino acids. Monocultures were transferred daily (1:100 dilution), whereas cocultures were transferred every three days for a total of 27 days. **B** Growth of Δ*lysA* and Δ*argH* strains in M9 medium under different amino acid supplementation regimes. Cultures were grown with the focal required amino acid (Lys or Arg), without amino acids (0), with the 19 non-focal amino acids (AAs), or with both the focal amino acid and the 19 other amino acids (Lys/Arg + AAs). Optical density (OD_600_) was measured after 24 h of cultivation. Different letters indicate significant differences (One-way ANOVA: Δ*argH*, *p* < 0.001, Δ*lysA*, *p* < 0.001). Population dynamics of Δ*lysA* (**C**) and Δ*argH* (**D**) monocultures during the evolution experiment. Each point represented a replicate, and dashed lines indicate mean population densities. Asterisks denoted statistically significant between groups (Paired samples *t*-test: *p* = 0.002). **E** Coculture density (Co) and each population density (Co-Δ*lysA* or Co-Δ*argH*) in cocultures dynamics over time (lines represent mean values). Asterisks denoted statistical significance between groups (Wilcoxon signed ranks test: *p* = 0.01). See also Supplementary Fig. S1. **F** Classification of coculture outcomes at the end of the experiment. Seven of ten cocultures showed extinction (inactive), whereas three maintained sustained growth (active). Relative abundances of Δ*lysA* and Δ*argH* in cocultures at the start (**G**) and end (**H**) of the experiment. Arrows indicate the three active cocultures. **I** Relative net growth of derived populations compared with their ancestral counterparts. Values represented the ratio of net growth of derived strains to that of ancestors under identical conditions. The dashed line indicated that the net growth of the derived and ancestral strains did not differ significantly. Different letters denoted significant differences among treatments (One-way ANOVA: *p* < 0.001 (Mono: *n* = 10, Inactive Co: *n* = 7, Active Co: *n* = 3)). Asterisks denoted statistically significant deviations of relative net growth from the ancestral baseline (one-sample *t*-test comparing a sample mean with 1: **p < 0.01, ***p < 0.001). See also Supplementary Fig. S2.

We revisited a well-established synthetic, mutually-beneficial interaction between lysine- and arginine-auxotrophic *E. coli* (Δ*lysA* and Δ*argH*)^23^. In a previous long-term evolution experiment with this system (similar to Fig. 1A), replicate cocultures maintained under identical conditions consistently diverged: most declined and lost detectable growth, whereas a minority sustained relatively stable densities^23^. This pattern of divergence suggests that community fate is determined by factors other than or in addition to those in the external environment or by evolutionary changes during coculture^24^. Instead, differences present among each ancestral strain at the time of community assembly may bias long-term outcomes.

In the absence of engineered overproduction of metabolites, we tested if variation in initial metabolite production predicts ecological persistence and evolutionary stability among auxotrophic Δ*lysA* - Δ*argH* consortia. We found that stable and unstable outcomes are reproducible and associated with differences in metabolite production present at the initiation of the experiment. We then measured trade-offs in growth, population decline, and susceptibility to invasion by non-metabolite producing mutants, identifying pre-existing phenotypic variation as a key determinant of syntrophic stability.

## Results

### Stability of auxotrophic cocultures

We first examined the growth responses of Δ*lysA* and Δ*argH* strains under different amino acid supplementation. Neither strain grew in M9 medium lacking its required amino acid. Supplementation with non-focal amino acids (300 µM of each) alone did not restore growth (Fig. 1B). Growth was restored by the addition of the required amino acid (300 µM) and further increased when the focal amino acid was supplied together with other amino acids (One-way ANOVA: Δ*lysA*, *p* < 0.001, *F* = 54015, df = 31; Δ*argH*, *p* < 0.001, *F* = 2523, df = 31, Fig. 1B). This showed that that once primary limitation is relieved, each auxotroph can also utilize additional metabolites to enhance population growth.

We next monitored population dynamics during a 27-day serial transfer experiment. Monocultures were transferred daily (27 transfers in total), whereas cocultures were transferred every 3 days over the same period (9 transfers; Fig. 1A). In monoculture, Δ*lysA* populations grew rapidly early and then remained relatively stable, whereas Δ*argH* monocultures showed a gradual but sustained increase in population density over time (Paired samples *t*-test comparing day 3 and day 27, Δ*lysA*: *t* = 1.89, *n* = 10, *p* = 0.092; Δ*argH*: *t* = 4.46, *n* = 10, *p* = 0.002, Fig. 1C, D). In contrast, cocultures showed heterogeneous dynamics among replicates (Fig. 1E and Supplementary Fig. S1). Total coculture density typically peaked within the first 3–6 days and then declined (Wilcoxon signed-ranks test comparing day 3 and day 27: *p* = 0.01, *Z* = −2.50, *n* = 10). At the end point of the experiment, seven of ten cocultures showed little or no detectable growth (extinction), whereas three maintained sustained population densities. We therefore classified cocultures as inactive or active, respectively (Fig. 1F). Changes in coculture density were accompanied by shifts in consortium composition. Although all cocultures were founded at similar strain ratios (Fig. 1G), inactive cocultures at day 27 were typically dominated by Δ*lysA*, whereas active cocultures retained a higher relative abundance of Δ*argH* (Fig. 1H). This observation directly validates the patterns reported in our foundational study^23^, confirming that Δ*lysA* dominance is a consistent ecological marker of failing syntrophic interactions in this system.

To assess whether these outcomes reflected evolutionary changes in growth performance in individual strains, we quantified net growth of derived populations relative to their ancestors. The derived Δ*lysA* strain, when in monoculture or active coculture showed no significant change in net growth compared to its ancestral baseline (One-sample *t*-tests: Mono-Δ*lysA*, *p* = 0.303, *t* = 1.09, *n* = 10; Active Co, *p* = 0.97, *t* = 0.047, *n* = 3; Fig. 1I and Supplementary Fig. S2). In contrast, inactive cocultures exhibited significantly reduced net growth (One-sample *t*-test: *p* < 0.001, *t* = 6.88, *n* = 7; Fig. 1I and Supplementary Fig. S2), whereas the Δ*argH* monoculture showed a significant increase over the course of evolution (One-sample *t*-test: *p* = 0.005, *t* = 3.66, *n* = 10; Fig. 1I). Together, these results demonstrate that syntrophic cocultures consistently diverge into active and inactive states with distinct population trajectories and compositions. Furthermore, we ruled out trace element depletion as a potential cause for the observed decline in coculture population growth. This is because a second round of coculture with conditioned medium from the first incubation showed similar growth dynamics, indicating that the trace elements in the M9 medium were sufficient to sustain cycle growth (Supplementary Fig. S3). Since these patterns cannot be explained by changes in monoculture growth alone, we next examined whether differences in metabolite exchange underlie the observed patterns in divergence.

### Metabolite production shapes coculture interaction dynamics

**Fig. 2:**
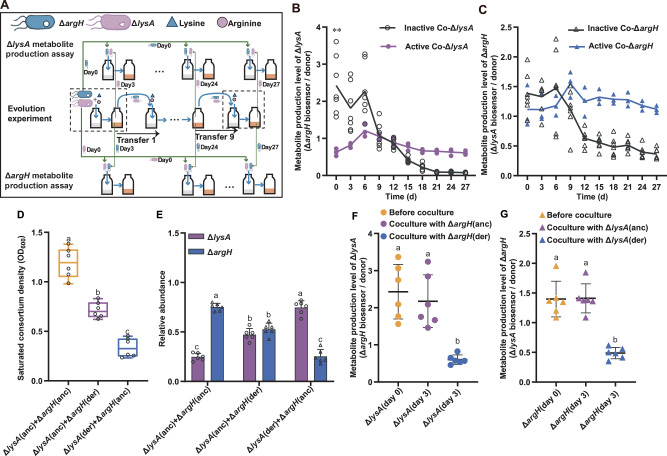
Temporal dynamics of metabolite production levels and consortium properties in auxotrophic cocultures. **A** Schematic diagram of the metabolite-production assay using biosensors during the evolution experiment. At specified time points, samples of the derived Δ*lysA* or Δ*argH* populations were cocultured with a complementary ancestral biosensor strain. Growth of the biosensor reflected the total pool of growth-supporting metabolites provided by the donor strain, including the focal amino acid and any additional accessible metabolites. To ensure consistency, the same ancestral Δ*lysA* (replicate No. 1) or Δ*argH* (replicate No. 1) isolate was used as the biosensor for all measurements. Per-cell metabolite production by Δ*lysA* (**B**) and Δ*argH* (**C**) lineages in active cocultures (filled symbol, *n* = 3) or inactive cocultures (open symbol, *n* = 7) over 27 days. Points denoted independent biological replicate, and the line indicated the mean values. The day 0 values represented the initial ancestral production levels. **D** Saturated consortium density (OD_600_) of 3-day reconstituted cocultures assembled from ancestral (anc) and/or derived (der) auxotrophs isolated from inactive cocultures: Δ*lysA*(anc)+Δ*argH*(anc), Δ*lysA*(anc)+Δ*argH*(der), and Δ*lysA*(der)+Δ*argH*(anc). Box plots show the medians and interquartile ranges; whiskers indicate the data ranges. **E** Relative abundances of Δ*lysA* (purple bars) and Δ*argH* (blue bars) in the corresponding 3-day reconstituted cocultures from (**D**). Bars showed mean values and s.d. Metabolite production levels of Δ*lysA* (**F**) and Δ*argH* (**G**) at day 0 (before coculture) and after 3-day coculture with either an ancestral or a derived partner strain. Different letters indicated statistically significant differences (One-way ANOVA, *p* < 0.001).

To quantify metabolites that each auxotroph made available to its partner, we employed a biosensor-based assay in which an ancestral recipient strain was cocultured with the focal donor strain (Fig. 2A). We chose this approach because standard supernatant measurements can underestimate effective exchange when shared metabolites are consumed or re-acquired near producer cells before they accumulate in the bulk medium^25^. This assay was motivated by the spatial proximity nature of exchange in our system. Metabolite exchange was significantly reduced when Δ*lysA* and Δ*argH* strains were separated by a membrane filter that allowed free diffusion of small molecules but prevented short-range cell-cell contact (Independent samples *t*-tests, *p* < 0.001, *t*_Δ*lysA*_ = 16.10, *t*_Δ*argH*_ = 21.33, *n* = 8, Supplementary Fig. S4A, B), which may result from metabolite re-acquisition by producer. Consistently, cell-free supernatants from monocultures of either auxotroph could not sustain the growth of the partner strain (Supplementary Fig. S4C), indicating that metabolite availability in coculture exceeds what is present in the bulk extracellular medium. These results show that spatial proximity between the auxotrophs enhanced effective cross-feeding. We therefore used biosensor growth as an integrated readout of shared metabolite, capturing contributions from both diffusible compounds and proximity-dependent exchange. Biosensor growth showed a linear increase across rising amino acid concentrations (Supplementary Fig. S5), confirming that this measure provides a quantitative proxy for metabolite production. Therefore, biosensor readings are presented as the effective metabolite production (number of biosensor cells fed per donor cell). To assess the reproducibility of biosensor-based metabolite-production phenotypes after frozen storage, we revived single-colony-derived ancestral Δ*lysA* and Δ*argH* isolates from frozen stocks after 0, 7, or 14 days of storage and then quantified metabolite production using the biosensor assay. Production levels were stable across storage durations, indicating that the assay captures a reproducible property of each isolate (Supplementary Fig. S6).

Using this assay, we monitored temporal changes in metabolite production during the evolution experiment. In inactive cocultures, metabolite production levels by both Δ*lysA* and Δ*argH* declined steadily with each transfer (Friedman’s repeated measures ANOVA: Δ*lysA*, *p* < 0.001, *χ*^2^ = 60.69, df = 9; Δ*argH*, *p* < 0.001, *χ*^2^ = 52.15, df = 9, Fig. 2B, C). By contrast, production levels of Δ*argH* in active cocultures remained relatively stable across transfers, while the Δ*lysA* showed slight production fluctuations (One-way repeated measures ANOVA: Δ*lysA*, *p* = 0.022, *F* = 12.56, df = 29; Δ*argH*, *p* = 0.243, *F* = 1.44, df = 29, Fig. 2B, C). Neither auxotrophic monoculture showed any significant change in metabolite production over the duration of the experiment (Paired sample *t*-tests: Δ*lysA*, *p* = 0.053, *t* = 2.231; Δ*argH*, *p* = 0.425, *t* = 0.836, *n* = 10, Supplementary Fig. S7). Notably, differences were evident from the outset: at day 0, active Δ*lysA* had significantly lower initial production than that of inactive Δ*lysA* (Independent samples *t*-test: *p* = 0.005, *t* = 3.87, *n*_inactive_ = 7, *n*_active_ = 3, Fig. 2B).

To test how changes in each strain following evolution experiment affected the interaction, we reconstituted cocultures with different combinations of ancestral and derived isolates (from day 27 inactive coculture of evolution experiment), and then measured their growth and metabolite production after 3 days. Consortia composed of two ancestral strains reached the highest cell densities, whereas any consortia containing a derived strain only grew to a significantly lower density (One-way ANOVA, *p* < 0.001, *F* = 83.52, df = 17; Fig. 2D). These differences aligned with shifts in consortium composition: the presence of a derived partner consistently and significantly increased the relative abundance of Δ*lysA* in the consortium (One-way ANOVA, *p* < 0.001, *F* = 90.71, df = 35; Fig. 2E). Moreover, when an ancestral strain was paired with its derived partner, the metabolite production of the ancestral strain dropped more sharply within 3 days than if it was paired with another ancestral strain (One-way ANOVA, Δ*lysA*: *p* < 0.001, *F* = 16.66, df = 17; Δ*argH*: *p* < 0.001, *F* = 31.7, df = 17, Fig. 2F, G).

**Fig. 3:**
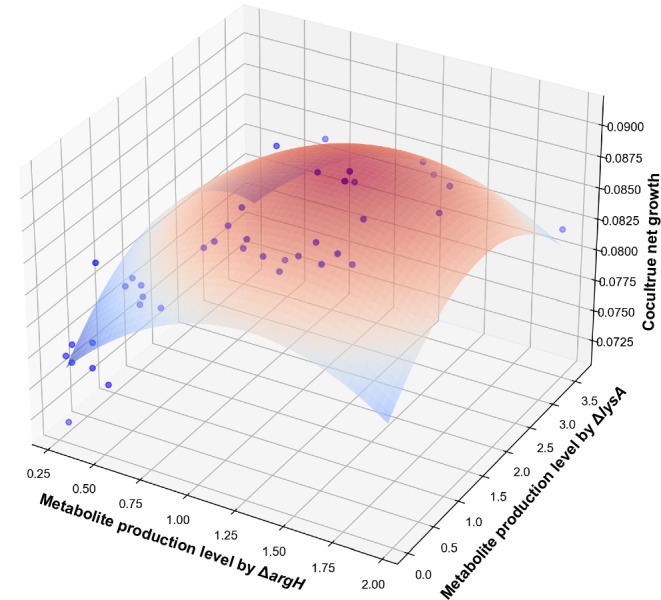
Metabolite production shows diminishing returns for consortium net growth. A curvilinear regression model was used to examine how metabolite production by Δ*lysA* and Δ*argH* jointly explain variation in coculture net growth. Experimental observations were shown as solid points, with the fitted surface indicated predicted net growth across the metabolite production of both strains. Net growth increased with metabolite output at low to intermediate levels but plateaued at higher production, consistent with diminishing net growth with surplus metabolite availability (*R*² = 0.847, *p* < 8.92 × 10^−14^, *F* = 48.3, *n* = 40). See also Supplementary Fig. S8.

A least-squares regression showed a significant curvilinear relationship between metabolite production and coculture net growth (*R*² = 0.847, *p* < 0.001, *F* = 48.3, df = 35, Fig. 3, Supplementary Fig. S8), with the metabolite production levels of both strains positively contributed to net growth up to a certain threshold, beyond which their marginal benefits diminished.

### Low initial metabolite production stabilizes syntrophy

**Fig. 4:**
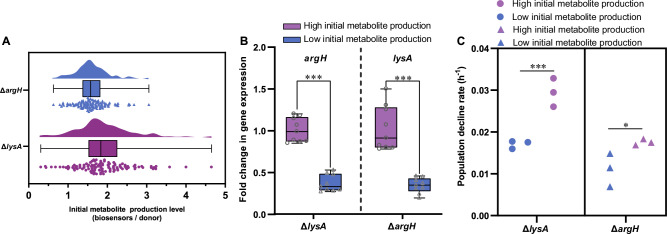
Variation in initial metabolite production and its association with gene expression and population decline. **A** Distribution of initial metabolite production levels among 100 independent single-colony isolates of ancestral Δ*argH* and Δ*lysA* strains, quantified using the biosensor assay. Violin plots showed the full distribution, with embedded box plots indicating the medians and interquartile ranges. **B** Fold changes in gene expression of *argH* or *lysA*, key genes involved in arginine or lysine biosynthesis, respectively. Asterisk indicated significant differences between groups (independent sample *t*-tests: ***p < 0.001). Points denoted three independent biological replicates, each comprising three technical replicates. Purple symbols represented high-production isolates, and blue symbols represented low-production isolates. **C** Population decline rates of Δ*lysA* and Δ*argH* strains with high versus low initial metabolite production. Each point represented an independent biological replicate. Statistical significance was shown (independent samples *t*-tests: *p < 0.05; ***p < 0.001). See also Supplementary Fig. S11.

We next asked whether variation in the founding strains’ metabolite production could predict coculture stability. Single-colony isolates obtained by streaking frozen glycerol stocks (from the same time period as the ancestral strain in the evolution experiment) showed wide variation in their initial metabolite production. Among 100 independent isolates of each auxotroph, production levels varied with coefficients of variation of ~40% for Δ*lysA* and ~25% for Δ*argH* (Fig. 4A). The distribution of production phenotypes was slightly right-skewed (skewness: Δ*lysA* = 0.65, Δ*argH* = 0.2) and leptokurtic (kurtosis: Δ*lysA* = 1.39, Δ*argH* = 0.55, Supplementary Fig. S9). This suggests that metabolite production in the ancestral strains is a highly variable trait.

To determine whether coding-region variation contributed to the observed production differences, we compared the coding-region sequences of high- and low-producing isolates using our current resequencing workflow, which was limited to annotated coding regions. Within the coding regions resolved by this analysis, we did not identify consistent differences between high- and low-production isolates of either strain (Supplementary Fig. S10). This analysis does not address non-coding variants, copy-number changes, rearrangements, or other structural differences and therefore does not exclude a genetic basis for the phenotype. Despite this uncertainty in molecular mechanism, quantitative PCR showed that high-producers expressed the relevant biosynthetic gene (*argH* in Δ*lysA*, or *lysA* in Δ*argH*) at significantly higher levels than low-producers (Independent samples *t*-tests: *argH*, *t* = 10.84, *n* = 9, *p* < 0.001; *lysA*, *t* = 7.27, *n* = 9, *p* < 0.001; Fig. 4B). Furthermore, we found that high-production isolates incurred significantly higher population decline rate than low-production isolates (Independent samples *t*-tests: Δ*lysA*, *t* = 6.06, *n* = 3, *p* = 0.004; Δ*argH*, *t* = 2.78, *n* = 3, *p* = 0.05; Fig. 4C; Supplementary Fig. S11). Together, these results show that the production phenotypes are associated with expression differences, but the underlying molecular basis remains unresolved.

Importantly, the production phenotype was neither purely fixed nor purely transient. Production differences were reproducible after regrowth from frozen single-colony isolates (Supplementary Fig. S6), and metabolite production remained similar to that of the ancestral strain in post-evolution monoculture controls (Supplementary Fig. S7). By contrast, in short-term coculture, in which ancestral strains were grown together with a derived low-production partner, the ancestral strains’ metabolite production dropped significantly within three days (Fig. 2F, G). This rapid change suggests that the production phenotype may largely be environmentally dependent and responsive to the microbial social context.

**Fig. 5:**
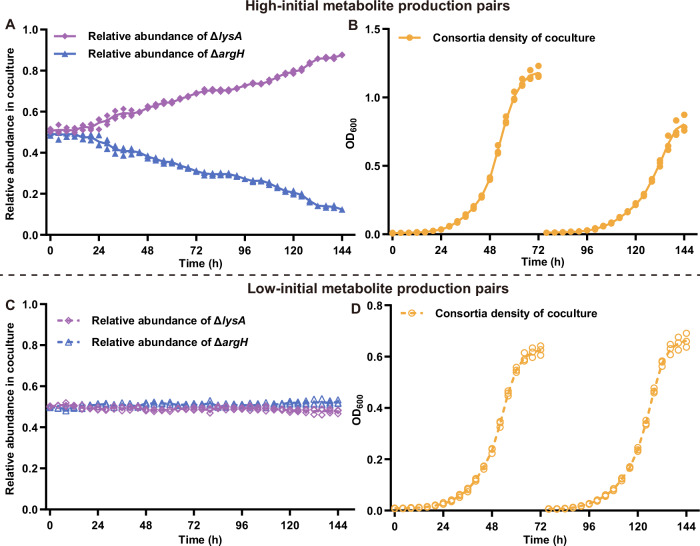
Consortium composition and growth dynamics across serial dilutions in cocultures founded by high- versus low-initial metabolite-production pairs. Cocultures were assembled from either high-initial metabolite production pairs (**A**, **B**) or low-initial metabolite production pairs (**C**, **D**). Cultures were grown for 72 h (Passage 1), diluted 1:100 into fresh M9 medium, and incubated for an additional 72 h (Passage 2). All assays were performed with three independent biological replicates (*n* = 3). **A** Relative abundances of Δ*lysA* (purple diamond) and Δ*argH* (blue triangles) at the Passage 1 and Passage 2 in cocultures founded by high-initial metabolite-production pairs. **B** Growth curves (OD_600_) of coculture and each strain during Passage 1 (0–72 h) and after 1:100 dilution during Passage 2 (72–144 h) for high-initial metabolite production pairs. **C** Relative abundances of Δ*lysA* and Δ*argH* at the Passage 1 and Passage 2 in cocultures founded by low-initial metabolite-production pairs. **D** Growth curves (OD_600_) of coculture and each strain during Passage 1 and Passage 2 for low-initial metabolite production pairs. Lines showed mean values across biological replicates (*n* = 3).

We then tested how initial levels of metabolite production influenced consortium stability. We assembled two pairs of cocultures using either two high-production isolates of each strain or two low-production isolates of each strain as founders, and subjected each to a dilution-regrowth cycle. After 72 h of growth, cultures were diluted 100-fold into fresh medium and then left for an additional 72 h (Fig. 5). Cocultures founded by high-production pairs showed pronounced shifts in relative abundance, where Δ*lysA* increased and Δ*argH* decreased in abundance (One-way repeated measures ANOVA: *p* < 0.001, *F* = 381.7, df = 37, Fig. 5A). The high-production consortia showed a significantly lower density in the second stationary phase than in the first (Friedman’s repeated measures ANOVA: 72 h vs144 h, *p* = 0.027, *χ*^2^ = 110.84, df = 37, Fig. 5B) following re-inoculation. In contrast, cocultures composed of low-production isolates regained their original composition (One-way repeated measures ANOVA: *p* = 0.094, *F* = 4.92, df = 37, Fig. 5C) and growth dynamics (Friedman’s repeated measures ANOVA: 72 h vs 144 h, *p* > 0.05, *χ*^2^ = 110.80, df = 37, Fig. 5D) after dilution, reaching similar densities of stationary phase to the first cycle. In extended serial transfers, only the low-production pairs maintained stable growth, whereas the high-production pairs eventually extincted (Supplementary Fig. S12). These findings demonstrate that consortia founded by low-producing strains can better withstand the negative effects of population bottlenecks than those founded by high-producing strains.

### Low-production consortia resist invasion by non-producers

**Fig. 6:**
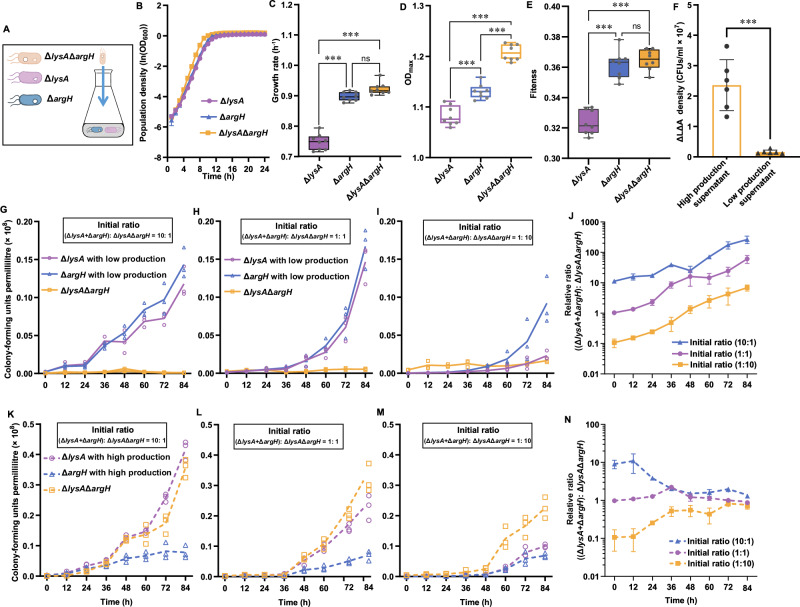
Non-producing mutants outcompeted high-production but not low-production cross-feeding consortia. **A** Schematic diagram of the invasion assay. A double auxotroph (Δ*lysA*Δ*argH*) was constructed and introduced into Δ*lysA* and Δ*argH* cocultures to test the susceptibility of consortia to competition from the introduced strain under different initial metabolite-production backgrounds. **B** Growth curves of Δ*lysA* (purple circles), Δ*argH* (blue triangles), and Δ*lysA*Δ*argH* (yellow squares) strains grown in monoculture supplemented with 300 μM lysine and 300 μM arginine (*n* = 8). **C** Maximum growth rates estimated from growth curves in (**B**). **D** Maximum optical densities (OD_max_) estimated from growth curves in (**B**). **E** Fitness of Δ*lysA*, Δ*argH*, and Δ*lysA*Δ*argH* strains quantified as the Malthusian parameter: ln (*N*_t_/*N*_0_) / 24, with *N*_0_ the initial CFUs count and *N*_t_ the final CFUs count (*n* = 8). **F** Growth of Δ*lysA*Δ*argH* in cell-free supernatants harvested from Δ*lysA* and Δ*argH* cocultures founded by high- or low-initial metabolite-production coculture pairs. Supernatants were sterile-filtered (0.22 μm) prior to inoculation. Time-course population densities (CFU/mL) of Δ*lysA*, Δ*argH*, and Δ*lysA*Δ*argH* during invasion assays conducted in low-initial metabolite-production cocultures (**G**–**I**) or in high-initial metabolite-production cocultures (**K**–**M**). Cocultures were initiated with different inoculation ratios of (Δ*lysA*+Δ*argH*): Δ*lysA*Δ*argH*: 10: 1 (**G**, **K**), 1: 1 (**H**, **L**), and 1: 10 (**I**, **M**). Lines showed mean values of three independent biological replicates (*n* = 3). Relative abundances of Δ*lysA*Δ*argH* during invasion assays in low-initial metabolite-production cocultures **J** or in high-initial metabolite-production cocultures (**N**), expressed as the ratio (Δ*lysA*Δ*argH*) / (Δ*lysA*+Δ*argH*) over time (corresponding to **G**–**I** or **K**–**M**). All points denoted independent biological replicates. For time-course panels (**B**, **J**, **N**), means and s.d. were shown. Statistical significance was indicated as shown in the figure (ns, not significant; ***p < 0.001).

In the evolution experiment, we observed that inactive cocultures became dominated by very low metabolite-production lineages, whereas active cocultures did not (Fig. 2B, C). This suggested that instability of a mutually beneficial interaction between strains may associate with the spread of reduced producers. To investigate further, we constructed a lysine-arginine double auxotroph (Δ*lysA*Δ*argH*) that cannot produce either amino acid, to represent a very low metabolite-production phenotype strain (Fig. 6A). Under nutrient-supplemented conditions, this double auxotroph exhibited higher fitness than the Δ*lysA* single auxotroph and similar fitness to Δ*argH* (One-way ANOVA: growth rate, *p* < 0.001, *F* = 160.6; biomass, *p* < 0.001, *F* = 115.1; fitness, *p* < 0.001, *F* = 65.8, df = 23; Fig. 6B–E). Thus, disabling amino acid production can be beneficial for a strain when metabolites are freely available in the environment, consistent with our previous findings that derived strains gained fitness advantages by reducing metabolite production in the early evolution stages^23^.

We next tested whether our metabolite non-producing strain could invade and outcompete a mutually beneficial interaction between two other strains. As a baseline, we grew Δ*lysA*Δ*argH* in cell-free supernatants from cocultures. This strain increased in numbers significantly more in the supernatant from high-production cocultures than in supernatant from low-production cocultures (Independent-samples *t* test, *p* < 0.001, *t* = 6.79, *n* = 6, Fig. 6F). This suggests that high-production cocultures generated a larger extracellular metabolite pool available to Δ*lysA*Δ*argH* strain. We then introduced the non-producer Δ*lysA*Δ*argH* strain into live low- or high-production cocultures at different initial frequencies. In low-production consortia, Δ*lysA*Δ*argH* consistently declined in abundance over time at all tested introduction frequencies (one-way repeated measures ANOVA, 10: 1, *p* = 0.01, *F* = 27.9; 1: 1, *p* = 0.03, *F* = 30.3; 1: 10, *p* = 0.02, *F* = 20.0, *n* = 3, Fig. 6G–J). By contrast, in high-production consortia, Δ*lysA*Δ*argH* persisted and in some cases increased in relative abundance (one-way repeated measures ANOVA, 10: 1, *p* = 0.08, *F* = 8.6; 1: 1, *p* = 0.01, *F* = 25.2; 1: 10, *p* = 0.03, *F* = 12.4, *n* = 3, Fig. 6K–N). These results show that although a completely non-metabolite producing phenotype can have a fitness advantage whilst in isolation, its ability to persist with other strains may depend on the wider metabolic environment.

Finally, because the pairing of high metabolite-production strains likely generated a larger pool of extracellular metabolites, and the production of metabolites will incur costs, we asked whether such environments actively select for a reduction in metabolite production. We therefore initially cultured both low- and high metabolite-producing isolates serially transferred for one month (transferring 1% of culture into fresh medium daily) in monoculture under either metabolite-rich (300 μM lysine and 300 μM arginine) or metabolite-poor (4 μM each) treatments. Populations derived from the high metabolite-producing isolates in the metabolite rich environment showed a marked decrease in metabolite production, which was reduced by approximately 25% in Δ*argH* and 40% in Δ*lysA* (Supplementary Fig. S13). In contrast, low metabolite-producing isolates showed no significant change in metabolite production regardless of treatment (Supplementary Fig. S13). Thus, once extracellular metabolites become abundant, selection favors reduced production primarily in lineages that initially invest more heavily in the trait.

## Discussion

We show that pre-existing variation in metabolite production at the time of community assembly can significantly affect the fates of syntrophic microbial consortia. In a simple syntrophic system composed of lysine- and arginine-auxotrophic *E. coli*, consortia founded by strains with lower initial metabolite production were more likely to maintain stable growth, whereas those founded by high-producing strains frequently became extinct. While experimental evolution clearly demonstrates that established mutualisms can evolve enhanced reciprocal exchange and deeper metabolic entanglement over time^22,26^. Our results reveal an additional constraint that excess metabolite production during the initial assembly of a consortium can actually undermine ecological stability.

Most experimental and theoretical work on mutually beneficial microbial interactions has focused on interaction structure, metabolic complementarity, and evolutionary change after systems have been established^8,27,28^. By contrast, less attention has been paid to the phenotypic variation that exists within strains or species before interactions occur. In our system, independently isolated colonies derived from the same engineered ancestor displayed continuous variation in metabolite production. This variation was sufficient to predict whether a consortium would persist or become extinct over tens of generations. Our resequencing analysis showed no consistent differences in coding regions between high- and low-production isolates that we examined, while expression of key biosynthetic genes differed significantly. These observations are consistent with differences in regulatory states, but could also relate to genetic differences outside coding regions that our analysis did not resolve. Because the metabolite production phenotypes were reproducible upon regrowth from single-colony isolates (Supplementary Figs. S6 and S7) yet also shifted under prolonged environmental treatment (Supplementary Fig. S13), the data suggest that these traits are partly stable across generations while remaining responsive to the environment. Determining whether that stability arises from unresolved genetic differences, epigenetic mechanisms, or other long-lived regulatory states will require additional work.

The reduced stability of high-production consortia most likely reflects a cascading failure of metabolic and ecological constraints. First, maintaining high metabolite production incurs a direct physiological burden, elevating the population decline rate of high-producing strains. Second, high production fundamentally changes the spatial economy of mutualistic exchange. When metabolite supply exceeds immediate local demand, these compounds accumulate in the environment, shifting exchange from a tightly localised process towards a diffusible public good^29^. This decoupling of production from mutualistic benefit makes the interaction vulnerable to exploitation and therefore unstable^30,31^. Our invasion experiments confirmed this dynamic: high-production environments allowed non-producing mutants to survive and accumulate, whereas low-production consortia restricted their growth. In addition, excess extracellular metabolites themselves create direct selection against the mutualism. Under metabolite-rich conditions, initially high-producing strains rapidly downregulate their production (Supplementary Fig. S13). Once one partner lowers its production, reciprocal supply also declines (Fig. 2F, G), triggering a rapid negative-feedback loop^32^ that eventually drives total population density below the threshold required to sustain cross-feeding^20^, leading to extinction. Given the intertwining of physiological costs, spatial leakage, and cheating dynamics, mathematical modeling will be a critical next step for disentangling these factors. Future theoretical frameworks should examine how the interplay among production burden, mutualistic benefit, and the initial frequencies of different founder phenotypes determines the boundary between stable coexistence and community collapse.

In contrast, low initial metabolite production promotes stable cross-feeding between strains by keeping metabolite production tightly matched to partner demand^33^. Under these conditions, metabolites were more likely to be consumed locally and less likely to accumulate as broadly accessible resources, making them more difficult for non-producers to access. This interpretation is consistent with our membrane-separation experiments and with theoretical work on partially privatized public goods^33^. In this sense, low production strengthens the mutualism not by providing more metabolites, but by localizing the benefits of production. Similar patterns have been observed in other microbial cooperative systems, suggesting that preventing a surplus production of metabolite may represent a general mechanism by which microbial mutually beneficial interactions remain stable^33,34^.

Our conclusion that low metabolite production enhances the stability of syntrophic consortia stems from studies on systems in which amino acid exchange plays a central role. In our system, amino acid exchange primarily occurs through a proximity-dependent mechanism. Whether these findings can be generalized to other systems largely depends on the characteristics of the exchanged metabolites and the spatial scale of interactions. For metabolites that rely on contact or exchange over short distances, low production will likely limit the distance that metabolites remain available to individual cells. This will restrict the benefits to neighboring cells, thus weakening any selective advantage to non-metabolite producing “cheaters”^35,36^. This is similar to how spatial structure and limited diffusion stabilize cooperation by forming positive pairings between producers and mutualist^37^. In contrast, for highly diffusible resources available in the wider environment, such as siderophores, certain vitamins, or signaling molecules, the spatial coupling between producers and beneficiaries is naturally weaker. For example, in *Pseudomonas-*mediated siderophore exchange, stable cooperation between partners often requires mechanisms beyond production regulation, such as spatial structuring, kin selection, and/or metabolic privacy^38,39^.

Several limitations of this study should be noted. First, our sequence comparison was limited to coding regions. We therefore cannot exclude causal differences in promoters or other non-coding regulatory elements, copy-number changes, genome rearrangements, or other structural features not resolved by the current analysis. Although the differences between high- and low-production isolates differ at the regulatory level, but whether those differences arise from uncharacterized genetic variants or from other heritable cellular states remains unresolved, which will be the focus of our future work. Second, our experiments examined a mutually beneficial interaction between two *E. coli* strains, whereas natural microbial communities typically involve multiple strains/species and metabolites. In such systems, phenotypic heterogeneity may interact with higher-order effects in ways that either amplify or buffer its effects. Third, microbial consortia can exchange multiple amino acids and other metabolites simultaneously^40^. Our current biosensor-based assay captures effective metabolite exchange but does not resolve the full spectrum of exchanged compounds. Future work will incorporate untargeted metabolomics and transcriptomics to provide a comprehensive view of metabolite flux and regulatory responses within cocultures.

In summary, our study shows that phenotypic variation for metabolite production in *E. coli* present at the onset of consortia assembly can bias the long-term stability of two-way syntrophic interactions. By linking initial levels of metabolite production to availability and feedback dynamics, our results help explain why many metabolically complementary systems fail to persist under laboratory conditions. Rather than favoring maximal metabolite production, a stable mutually beneficial interaction emerges when metabolite exchange is constrained, tightly coupled, and resistant to over-exploitation by partners. These principles may enhance our understanding of both the interpretation of natural microbial communities and the design of synthetic consortia.

## Methods

### Experimental *E. coli* strains and culture condition

All experiments used *E. coli* MG1655 lysine and arginine auxotrophs (Δ*lysA* and Δ*argH*), generated by CRISPR-Cas9-mediated knockout of *lysA* and *argH*^23^. Strains were distinguished using plasmid-borne fluorescent markers (GFP/mCherry). We found previously that fluorescent plasmid effects on growth were negligible under our current experimental conditions^23^. A double auxotroph (Δ*lysA*Δ*argH*) was constructed by deleting *argH* in the Δ*lysA* background using the same CRISPR-Cas9 protocol^23^. Unless otherwise stated, all auxotrophic strain cultures were grown in M9 minimal medium (6.8 g/L Na_2_HPO_4_, 3 g/L KH_2_PO_4_, 1 g/L NH_4_Cl, 0.5 g/L NaCl, 2 mM MgSO_4_·7H_2_O, 0.5 mM thiamine hydrochloride, 0.0025 g/L FeSO_4_·7H_2_O, and 0.5% (wt/vol) glucose). Cultures were incubated at 37 °C whilst shaking at 220 rpm in 12 mL sterile culture tube (Sangon Biotech) containing 5 mL of M9 medium. Kanamycin (50 µg/mL, Sangon Biotech) was included to maintain fluorescent plasmids. Population densities were assessed based on optical density readings (Biotek Synergy Neo2, Vermont, America) at 600 nm (OD_600_) or determined by colony-forming units per milliliter (CFU/ml) on selective M9 agar plates supplemented with either lysine or arginine (150 µM). For CFU assay, the culture was serially diluted in sterile M9 medium, and 100 μL of each dilution was inoculated onto three separate M9 agar plates. Plates with 30-300 colonies were selected for CFU determination.

### Metabolic flexibility of auxotroph assay

To assess the metabolic flexibility and auxotrophic compensation of engineered *E. coli* strains, we implemented a flexible nutrient supplementation assay. Specifically, we evaluated whether auxotrophic strains could compensate for missing metabolites through alternative amino acids within their metabolic network. For each auxotrophic strain, growth was evaluated under four conditions: (i) supplementation with the target amino acid (300 μM lysine or arginine in M9 medium; positive control); (ii) no amino acid supplementation (negative control); (iii) supplementation with each of the other 19 proteinogenic amino acids at 300 μM (excluding the target amino acid), to probe potential biochemical conversion to the missing metabolite; and (ⅳ) combined supplementation with the target amino acid (300 μM lysine or arginine) and all other 19 amino acids (each at 300 μM). For each condition, eight independent colonies (*n* = 8) from each auxotrophic strain (Δ*lysA* and Δ*argH*) were tested. The precultures were established by inoculating individual colonies into M9 medium and incubating overnight at 37 °C with shaking at 220 rpm. Cultures were then diluted into 5 ml fresh M9 medium to an initial OD_600_ ~ 0.005 and grown for 24 h under the same conditions. Growth was quantified by measuring optical density at 600 nm using a microplate reader (BioTek Synergy Neo2, VT, USA).

### Laboratory evolution

To standardize baseline amino acid availability across treatments, we first determined an amino-acid supplementation level that would produce a ~2-fold overnight increase in amount of growth from an initial level of OD_600_ ~ 0.005. Both auxotrophs reached this threshold with 4 µM lysine or arginine (Supplementary Fig. S14). This concentration was therefore used as the upper limit for amino acid supplementation for experimental evolution. Equal amino acid concentrations were supplied to monocultures and cocultures to ensure consistent nutrition across treatments. This was chosen as a conservative operational threshold to reduce the likelihood that excess amino acids would mask interactions between strains in coculture^41^.

The experiment involved three treatment groups: groups 1 and 2 were monocultures of each Δ*lysA* or Δ*argH*, grown in M9 medium supplemented with a minimum concentration (4 μM) of the appropriate amino acid, respectively. Group 3 was a coculture of both Δ*lysA* and Δ*argH* in M9 medium supplemented with 4 μM lysine and 4 μM arginine (Fig. 1A). For each strain, ten independent ancestral colonies (*n* = 10) were isolated from frozen glycerol stocks on lysozyme broth (LB) kanamycin agar and pre-cultured overnight in M9 supplemented with 300 µM of the appropriate amino acid. These pre-cultures were then washed three times with M9 medium, diluted to OD_600_ ~0.5, and inoculated at 1% (v/v). Each isolate was used to initiate one monoculture and one coculture. Each monoculture and coculture therefore shared the same ancestral origin, with each of the ten replicates founded by a distinct ancestral colony or colonies.

Each coculture was initiated using the same number of cells for each strain (Δ*lysA*: (4.57 ± 0.51) × 10^5^, Δ*argH*: (4.73 ± 0.32) × 10^5^ cells/ml) at the twice the total density as for a monoculture. All cultures were incubated at 37 °C, with constant shaking at 220 rpm. Based on the respective growth curves of the cultures^23^, monocultures were transferred daily (1:100 (v/v)) into 5 mL fresh medium, whereas cocultures were transferred every three days (1:100), with CFU/ml determined at each transfer stage. Generations per cycle were calculated as *g* = ln (*N*_t_/*N*_0_)/ln (2), with N_0_ the initial cell density and *N*_t_ the final cell density for a given growth cycle. This procedure spanned a total of 27 days, equating to approximately 55 generations (summed across cycles) in cocultures and 170 generations in monocultures. At each coculture transfer, populations were plated on selective media to isolate each strain. Three colonies per strain per replicate were stored (15% glycerol, −80 °C) for downstream assays. Following laboratory evolution, we tested whether the auxotrophic strains had reversed to the prototrophic phenotype^42^. By inoculating derived colonies on amino acid-free M9 agar plates and M9 agar plates supplemented with lysine or arginine at 37 °C for 24 h, we found that strains in either coculture or monoculture remained auxotrophic by the end of the experiment.

### Relative net growth assay of ancestral and derived populations

To determine if and how the growth of the derived populations changed after laboratory evolution, we measured the net growth of the derived auxotrophic strains (Evo) relative to the corresponding ancestral strains (Anc). For monocultures (*n* = 10), ancestral or derived strains were inoculated into M9 supplemented with 4 µM of the appropriate amino acid and incubated for 24 h (37 °C, 220 rpm). For cocultures (*n* = 3 for active coculture, *n* = 7 for inactive coculture), ancestral-ancestral or derived-derived cocultures were initiated without amino-acid supplementation and incubated for 72 h. CFUs were determined at 0 h and at the endpoint. Relative net growth was calculated as: relative net growth = ln (*N*_t, Evo_/*N*_0, Evo_)/ln (*N*_t, Anc_/*N*_0, Anc_), with *N*_0_ the initial CFU count and *N*_t_ the final CFU count^22^.

### Quantification of metabolite production levels using biosensors

To quantify metabolite exchange between auxotrophic partners, we used an established growth-based biosensor assay^22,43^. In such an assay, the biomass accumulation of an auxotrophic recipient strain serves as an integrative readout of metabolite availability supplied by a cocultured donor, because recipient growth is strictly dependent on metabolites released or made accessible by the partner strain^22,43^. Because recipient growth can be supported not only by the focal amino acid but also by additional metabolites once the primary limitation is relieved (Fig. 1B, see Results), biosensor output can be interpreted as net growth-supporting metabolite availability rather than a strictly single-metabolite measurement. The fixed Δ*lysA* or Δ*argH* single colony was used as a biosensor and cocultured with strains requiring metabolite quantification (i.e., donors). One strain was therefore the biosensor of the other when in coculture (Fig. 2A).

In practice, 50 μl of each diluted pre-culture of donor and biosensor were cocultured in 5 ml of M9 medium containing the appropriate amino acid (300 μM) required by the donor strain for 72 h at 37 °C, whilst being stirred at 220 rpm. The resultant value of metabolite production for each donor cell was normalized by dividing the CFU of the biosensor strain cells by the CFU of the donor strain cells after the 72 h time period (metabolite production level = CFU_(biosensor)_/CFU_(donor)_). This controlled for the likely positive correlation between donor cell growth and biosensor growth, and provides a comparative measure of how many biosensor cells can be supported per donor cell. All assays were performed with three (*n* = 3, isolated from active coculture) or seven (*n* = 7, isolated from inactive coculture) independent biological replicates.

### Stability assay of syntrophic consortia

Following the methods described by Aulakh et al. to assess the stability of metabolically interdependent consortia^11^, stability in this study was operationally defined as the ability of a coculture to recover its population density and consortium composition following serial transfers (at volume of 1:100). We first randomly selected 100 independent Δ*lysA* colonies and 100 independent Δ*argH* colonies (in ancestral stage) and then identified their initial metabolite production levels by the biosensor method described above. From each strain, we then selected three low-production (Δ*lysA*: 0.28 ± 0.041, 0.40 ± 0.013, 0.41 ± 0.007; Δ*argH*: 0.60 ± 0.019, 0.77 ± 0.024, 0.81 ± 0.013 biosensors/donor) and three high-production isolates (Δ*lysA*: 3.22 ± 0.068, 3.58 ± 0.152, 4.00 ± 0.078; Δ*argH*: 2.20 ± 0.068, 2.23 ± 0.150, 2.56 ± 0.053 biosensors/donor) for resequencing, and assembled matched cocultures (low-low and high-high). Isolates were pre-cultured overnight in M9 with 300 µM of the required amino acid, washed three times in M9, adjusted to OD_600_ ~ 0.5, and co-inoculated (50 µL each) into 5 mL M9 supplemented with 4 µM lysine and 4 µM arginine. Cocultures were incubated for 72 h (Passage 1) at 37 °C whilst shaking at 220 rpm, diluted 1:100 into fresh medium, and incubated for a further 72 h (Passage 2) at same conditions, for a total of 144 h. OD_600_ was measured every 2 hours by the reading of optical density. Strain ratios were quantified by a confocal laser scanning microscope (Nikon AX, Japan) equipped with a 20× objective lens, which had been previously shown to reliably detect the relative abundances of strains (mCherry for Δ*lysA* and GFP for Δ*argH*)^23^. Samples were diluted ~100-fold in M9 and then imaged (field of view 880 × 880 µm). Cells were counted using ImageJ software (v1.53 s)^44^ with threshold-based segmentation and particle analysis (minimum size 5 pixels). For each time point, three imaging replicates were collected to account for spatial heterogeneity. Three independent biological replicates (*n* = 3) were performed.

### RNA extraction and cDNA synthesis

Total RNA was extracted from Δ*lysA* and Δ*argH* strains derived from three single colonies (*n* = 3) exhibiting either high or low initial metabolite production. Precultures were grown in M9 medium supplemented with 300 μM lysine or arginine at 37 °C with shaking at 220 rpm for 12 h. Cells were harvested by centrifugation at 12,000 rpm for 5 min at 4 °C and lysed using RNAiso Plus reagent (Takara) according to the manufacturer’s instructions. Briefly, cell pellets were resuspended in 500 μl RNAiso Plus and stored at −80 °C for 1 h. Samples were then centrifuged at 12,000 rpm and 4 °C for 5 min, and the supernatant was collected. Following the addition of 100 μl chloroform and mixing, 500 μl isopropanol was added. After centrifugation at 12,000 rpm for 15 min at 4 °C, RNA was precipitated and washed twice with 75% ethanol. The resulting RNA pellet was resuspended in RNase-free water and treated with DNase I (RNase-free, Thermo Fisher Scientific) to remove residual genomic DNA. RNA concentration and purity were determined using a NanoDrop spectrophotometer (Thermo Fisher Scientific), and samples with A260/A280 ratios close to 2.0 were used for downstream applications. For cDNA synthesis, 500 ng of total RNA was reverse-transcribed using the GoScript™ Reverse Transcription Mix (Promega) following the manufacturer’s instructions. Briefly, RNA was mixed with 4 μl Reaction Buffer and 2 μl Enzyme Mix, and nuclease-free water was added to a final volume of 20 μl. The reaction was incubated at 42 °C for 20 min, followed by enzyme inactivation at 85 °C for 5 min and immediate cooling on ice. The resulting cDNA was stored at −20 °C for subsequent quantitative PCR analysis.

### Quantitative PCR (qPCR)

Quantitative PCR was employed to quantify the relative expression levels of key biosynthetic genes associated with lysine and arginine production in auxotrophic strains. In Δ*argH*, lysine biosynthesis was evaluated by assessing the expression of *lysA* (encoding meso-diaminopimelate decarboxylase, which catalyzes the terminal step in lysine synthesis)^45^. In Δ*lysA*, arginine biosynthesis was examined by quantifying *argH* (encoding the enzyme that catalyzes the final conversion of argininosuccinate to arginine)^46^. The housekeeping gene *gapA* served as an internal reference for normalization. Primers were designed using Primer3 (http://primer3.ut.ee/) with the following sequences:

*lysA*: forward 5′-CGTTAATCCGGGGTTTGGTC-3′, reverse 5′-TCAACGCCAGAACCAATGTG-3′.

*argH*: forward 5′-TTACCCAGGCAGCAGATCAA-3′, reverse 5′-GCGAACATCTTCCAGCAACA-3′

*gapA*: forward 5′-GAAATGGGACGAAGTTGGTG-3′, reverse 5′-AACCACTTTCTTCGCACCAG-3′

qPCR reactions were conducted in a final volume of 10 μL, comprising 5 µL SYBR Green Master Mix (QIAGEN), 0.7 µL of each primer (final concentration of 0.7 µM), 1 µL of cDNA template, and 2.6 µL of RNase-free water. All reactions were performed in triplicate using an Applied Biosystems QuantStudio 5 real-time PCR system (Thermo Fisher Scientific). The amplification conditions included an initial denaturation at 95 °C for 2 min, followed by 40 cycles of 95 °C for 5 s and 60 °C for 10 s. Threshold cycle (CT) values were used for gene expression analysis. Relative changes in gene expression levels were determined using the 2^−ΔΔCT^ method^47^, with high initial metabolite production strains serving as the reference baseline. Three independent biological replicates (*n* = 3) were performed.

### Population decline rate assay

To quantify population decline rate under amino acid starvation, auxotrophic strains were subjected to a starvation assay designed to deplete intracellular amino acid reserves. Overnight precultures were washed three times with amino acid-free M9 medium and then incubated in the same medium for 4 h to exhaust residual metabolites. All cells were subsequently inoculated into fresh M9 medium without amino acid supplementation and cultured at 37 °C with shaking at 220 rpm for 24 h. Colony density was determined every 2 h by plating for colony-forming units. Changes in colony density during starvation were used to estimate the decline in population size, which reflects the combined effects of cell death and residual cell division. The natural logarithm of population density (ln(CFU/ml)) was regressed against time: *ln(N*_*t*_*)* = *ln(N*_*0*_*)* + *m · t*, where *N*_*0*_ the initial CFUs count, *N*_*t*_ the final CFUs count, and *t* the time. The slope (*m*) of the linear regression was used as an estimate of the population decline rate (per hour). More negative values of *m* indicate faster population decline. For ease of interpretation, the corresponding decline rates in Fig. 4C are plotted as positive values (-*m*). Three independent biological replicates (*n* = 3) were performed.

### Genome resequencing and variant analysis

To identify genetic differences associated with variation in metabolite production, we performed genome resequencing of selected isolates of Δ*lysA* and Δ*argH*. Three independent single-colony isolates exhibiting high initial metabolite production and three isolates exhibiting low production were selected for each strain (*n* = 3). Genomic DNA was extracted from overnight cultures grown in M9 medium supplemented with the required amino acid (300 μM) using a Bacterial DNA Extraction Kit (FINDROP, Guangzhou, China) according to the manufacturer’s protocol. DNA quality and concentration were assessed using a Qubit 4.0 Fluorometer (Life Technologies) and a Qsep400 system (Houze Biological Technology). Sequencing libraries were prepared using the ALFA-SEQ DNA Library Prep Kit (FINDROP) following standard protocols. Paired-end sequencing (150 bp) was performed on an Illumina NovaSeq 6000 platform (Guangdong Magigene Biotechnology Co., Ltd.). Raw reads were subjected to quality control by removing adapter sequences and low-quality reads using standard filtering procedures. Reads with Phred quality scores below Q20 were excluded, and overall data quality was evaluated based on Q20 and Q30 metrics. Clean reads were aligned to the Escherichia coli MG1655 reference genome (GenBank accession: GCF_000005845.2) using BWA-MEM (v0.7.17) with default parameters. SAMtools (v1.7) was used to convert, sort, and index alignment files. Mapping quality and genome coverage were assessed to ensure reliable alignment. Single nucleotide polymorphisms (SNPs) and small insertions/deletions (indels) were identified using SAMtools mpileup and bcftools with standard filtering criteria. Variants were retained only if they met the following criteria: minimum mapping quality ≥30, minimum read depth ≥10, and consistent support from both forward and reverse reads. Variant calling in this study was restricted to annotated coding regions. Non-coding variants, copy-number variations, and large structural rearrangements were not systematically analyzed. Functional annotation of variants was performed based on gene annotations of the reference genome. Sequencing depth and coverage were further evaluated by calculating the proportion of the genome covered at ≥1×, ≥10×, ≥50×, and ≥100× depth to ensure sufficient resolution for variant detection.

### Statistics and reproducibility

Normality and homogeneity of variances were assessed using Shapiro-Wilks and Levene’s tests, respectively. To evaluate differences in the means of two non-independent groups, such as growth changes before and after experimental evolution, we used paired t-tests or Wilcoxon signed ranks test. One-sample t-tests were used to compare the relative fitness of derived strains to their ancestral strain, which was set to 1. One-way analysis of variance (ANOVA) followed by Bonferroni post-hoc pairwise comparisons was used to test differences in means between three or more independent groups. When normality and variance homogeneity tests met the assumptions of parametric tests, one-way repeated measures ANOVA was used to assess the differences among non-independent groups across multiple time intervals, with *p*-values adjusted by Bonferroni correction^48^. Otherwise, Friedman’s repeated measures ANOVA on ranks test was used. A significance threshold of *α* = 0.05 was applied for all statistical tests. Skewness and kurtosis were used to describe distributions of metabolite production levels. These statistical tests were performed using SPSS 25.0.

To assess the effects of metabolite production levels by Δ*lysA* and Δ*argH* cells on coculture net growth, a second-order nonlinear least-squares regression model was constructed. Metabolite production levels of both strains were treated as independent explanatory variables in the model. Metabolite production was quantified using the biosensor assay and shown in Fig. 2, and net growth values were obtained from consortia sampled at day 0, day 3, day 15, and day 27 (Supplementary Fig. S2). This model was chosen based on observed curvilinear relationships between metabolite production and coculture net growth and was expressed as:

$${{\rm{Coculture}}}\; {{\rm{net}}}\; {{\rm{growt}}}{{\rm{h}}}=a+b\cdot {{\rm{Lys}}}+c\cdot {{\rm{Arg}}}+d\cdot {{\rm{Ly}}}{{{\rm{s}}}}^{2}+e\cdot {{\rm{Ar}}}{{{\rm{g}}}}^{2},$$

where *a* is the intercept, *b*, *c*, *d*, and *e* are the regression coefficients. Model parameters were estimated using the *curve_fit* function from the *scipy.optimize* module in Python 3.13. A 3D surface plot was generated using *matplotlib* to visualize the fitted surface and experimental data points. A summary of the results for this model is presented in Table S1.

### Reporting summary

Further information on research design is available in the Nature Portfolio Reporting Summary linked to this article.

## Supplementary information

Transparent Peer Review file

Supplementary Information: Low initial metabolite production enhances stability in syntrophic bacterial consortia

Reporting Summary

## Data Availability

Data used in this manuscript are available on Figshare^49^: 10.6084/m9.figshare.29821316. Additional genomic data generated in this study have been deposited under the BioProject PRJNA1233764 in the NCBI database.

## Code availability

Code used in this manuscript is available on Figshare: 10.6084/m9.figshare.29821316.
