# Supplementary material for: Low initial metabolite production enhances stability in syntrophic bacterial consortia
Source: Commun Biol. 2026 May 6;9:938. doi: 10.1038/s42003-026-10187-y (PMC13350956; doi:10.1038/s42003-026-10187-y)
Supplement: Supplementary file 2 — Supplementary Information: Low initial metabolite production enhances stability in syntrophic bacterial consortia [file 42003_2026_10187_MOESM2_ESM.pdf]

Low initial metabolite production enhances stability in syntrophic bacterial consortia

Nan Ye[1,2,3], Derek W Dunn[4], Zhichun Yang[3], Beibei Hou[3], Huan Wang[3], Jianxiao Song[3*], Rui-Wu Wang[5*]

1. Center for Materials Synthetic Biology, Shenzhen Institute of Synthetic Biology, Shenzhen Institute of Advanced Technology, Chinese Academy of Sciences, Shenzhen 518055, China

2. State Key Laboratory of Quantitative Synthetic Biology, Shenzhen Institute of Synthetic Biology, Shenzhen Institutes of Advanced Technology, Chinese Academy of Sciences, Shenzhen, China

3. School of Life Sciences and Technology, Northwestern Polytechnical University, Xi'an, 710072, China

4. College of Life Sciences, Northwest University, Xi'an, Shaanxi, 710069, China

5. College of Life Sciences, Zhejiang University, Hangzhou, China

*Correspondence: sjx@nwpu.edu.cn (J.S.); wangrw@zju.edu.cn (R.W.)

Table S1. Estimated parameters from the nonlinear quadratic model describing the relationship between metabolite production and coculture net growth improvement. Negative coefficient values for the quadratic terms indicate a concave surface and diminishing returns.

Parameter	Coefficient	Standard error	t-statistic	p-value	95% confidence interval (lower)	95% confidence interval (upper)
Intercept	0.0710153	0.00167	42.584	9.61×10^{-14}	0.06763	0.074401
Lys	0.0195005	0.00480	4.0598	0.000263	0.009749	0.029252
Arg	0.0068226	0.00191	3.568	0.001067	0.002941	0.010705
Lys^2	-0.00777	0.00221	-3.518	0.001225	-0.01225	-0.00329
Arg^2	-0.001798	0.00054	-3.360	0.001894	-0.00289	-0.00071

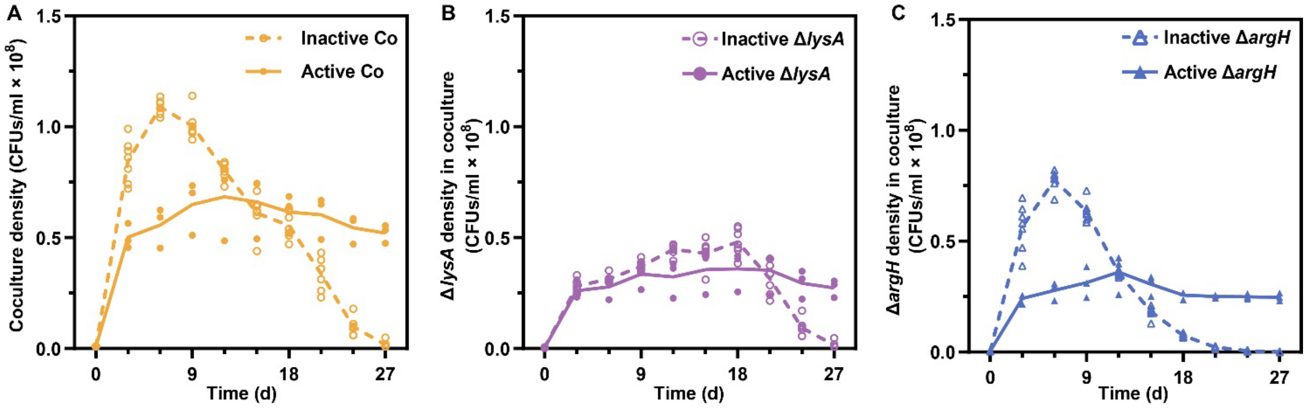

Fig. S1. **Population density dynamics of auxotrophic strains during coculture in the active groups versus the inactive groups in laboratory evolution experiments.** (A) Comparison of density changes in active consortia (yellow solid line with filled circles) and inactive consortia (yellow dashed line with open circles). (B) Comparison of density changes of Δ*lysA* population in active cocultures (purple solid line with filled circles) and inactive cocultures (purple dashed line with open circles). (C) Comparison of density changes of Δ*argH* population in active cocultures (blue solid line with filled triangles) and inactive cocultures (blue dashed line with open triangles). All data showed as mean and s.d. for the three biological replicates in the active group or the seven biological replicates in the inactive group.

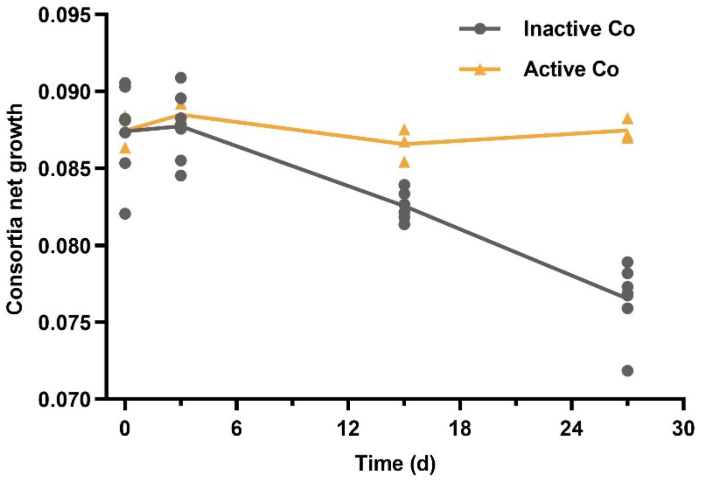

Fig. S2. **Consortia net growth dynamics of coculture over the course of the evolution experiment**. We isolated single colonies from each coculture at different time points over the duration of evolution experiment and used to re-establish cocultures under standardized conditions. Consortia net growth was quantified as the log-transformed fold change in total coculture cell density over the incubation period: ln $(N_t / N_0) / 72$, with N_0 the initial coculture CFUs count and N_t the final coculture CFUs count[1]. Active cocultures (yellow solid line with filled triangles) maintained net growth levels (One-way repeated measures ANOVA: $F = 2.11$, $df = 11$, $p = 0.20$, $n = 3$), whereas the inactive cocultures (grey solid line with filled circles) significantly decreased in net growth (One-way repeated measures ANOVA: $F = 35.21$, $df = 27$, $p < 1.67 \times 10^{-4}$, $n = 7$). Points represent independent replicates.

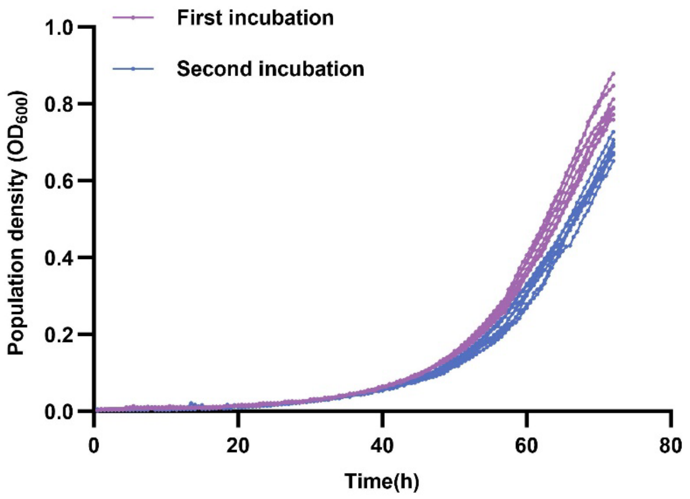

Fig. S3**. Trace elements in M9 medium did not limit coculture growth across serial incubations**. The growth curves of the coculture in the first incubation (purple) and second incubation (blue) were measured. After centrifugation and filtration of the coculture supernatant, the supernatant was used for a second round of coculture with fresh cells (the same amount of starting ancestral cells as in the first round) without adding any amino acids or glucose. Minor difference was observed between the first and second incubations, indicating that the trace elements in the M9 medium were sufficient to support coculture growth during a cycle (3 days) in the evolutionary experiment. Each line represents an independent biological replicate.

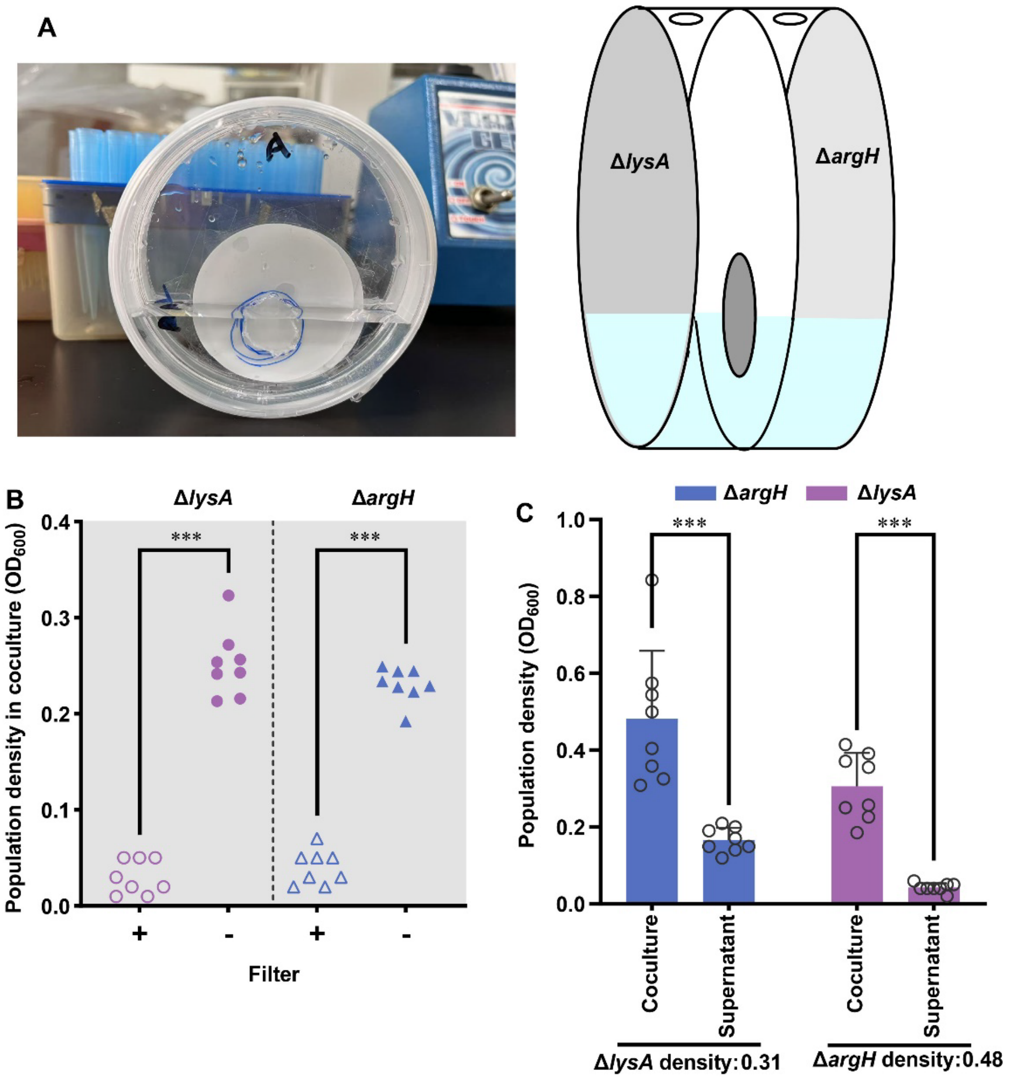

Fig. S4. **Metabolite exchange at short spatial proximity in cocultures.** (A) A device that allows cultivating two bacterial populations separated by membrane filters. The cells were assembled from two 90 mm petri dishes, each with a small hole in the bottom, joined together with sealing tape. The white disc in the middle (gray circle in the middle) represents the filter membrane (0.22 µm). (B) Short-range metabolite exchange. Δ*lysA* and Δ*argH* strains were either cocultured in the same compartment without a filter membrane (- Filter) or separated by a filter membrane (+ Filter) that allowed free metabolite to pass through but prevented direct physical contact between cells. Growth over 72 hours was determined by optical density (OD_{600}), comparing OD_{600} values of the same strain with and without the filter membrane. Asterisks indicate significant differences (independent samples t-tests, $p < 0.001$, $t_{\Delta lysA} = 16.10$, $t_{\Delta argH} = 21.33$, $n = 8$). (C) The metabolite secreted into the supernatant from the monoculture of the auxotrophic strain are insufficient to sustain the growth of the partner strain. Both Δ*lysA* and Δ*argH*, with an initial OD_{600} of 0.01, were cocultured in M9 medium devoid of amino acid supplementation for 72 hours. The Δ*lysA* strain achieved an average OD_{600} of 0.31 (±0.08), while the Δ*argH* strain reached 0.48 (±0.17). In contrast,

when Δ*lysA* was monocultured in M9 medium supplemented with 300 μM lysine to an OD_{600} of 0.31, the supernatant was filtered through a 0.22 μm membrane, mixed with 2×M9 at a volume of 1:1 ratio, and subsequently inoculated with Δ*argH*, initially at an OD_{600} of 0.01. After 24 hours, the Δ*argH* strain density was measured. The same procedure was applied to determine the extent to which the supernatant from Δ*argH* monoculture could support Δ*lysA* strain growth. Statistical significance, indicated by the asterisk, shows that the strain density supported by the supernatants of the two auxotrophic strains is substantially lower than the strain density achieved through physical proximity in cocultures (independent samples t-tests, $p < 0.001$, $t_{\Delta argH} = 4.98$, $t_{\Delta lysA} = 8.58$, $n = 8$).

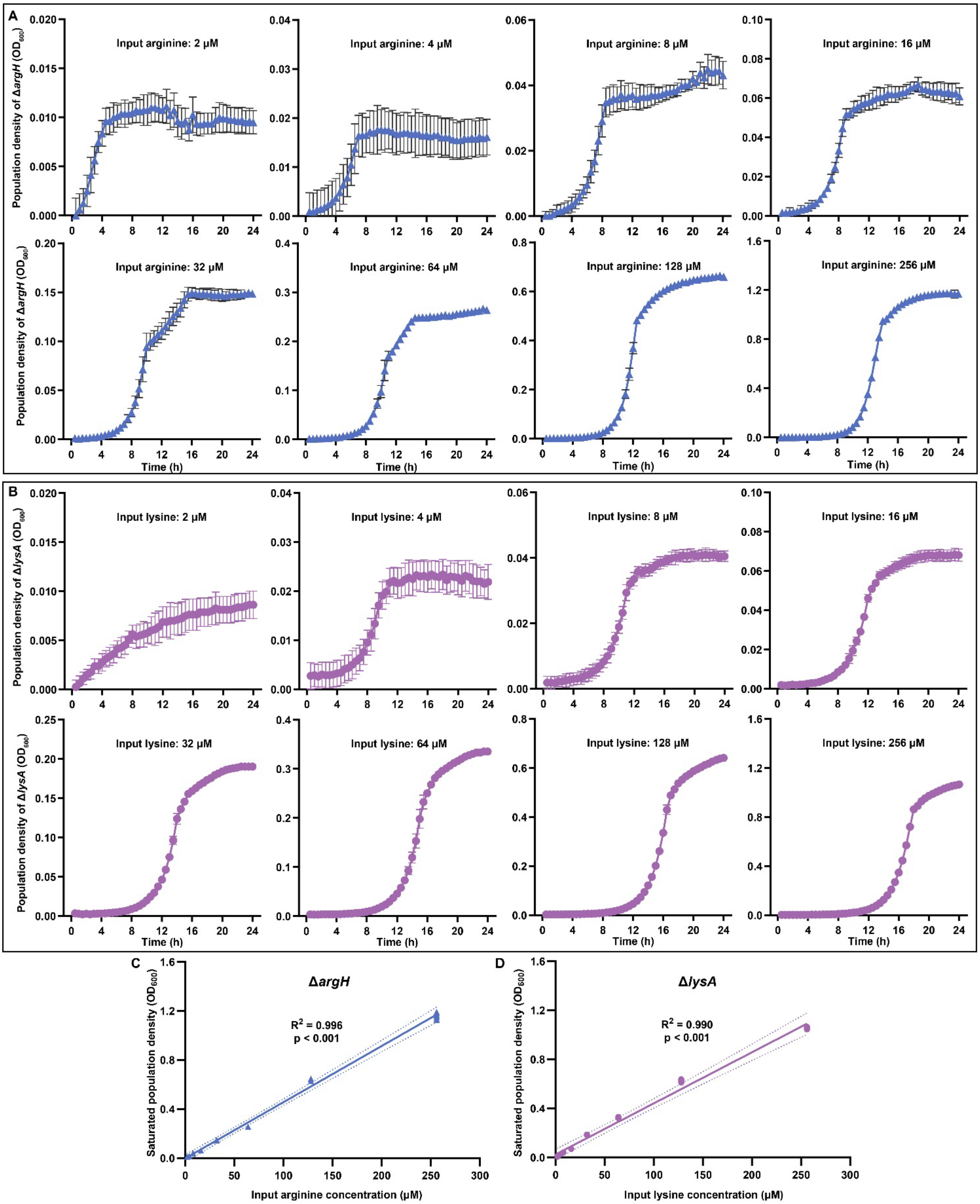

Fig. S5. **The growth of biosensors is linearly proportional to amino acid availability.** (A) Growth curves of the Δ*argH* strain in M9 medium supplemented with different arginine concentrations (2, 4, 8, 16, 32, 64, 128, 256 μM). (B) Growth curves of the Δ*lysA* strain in M9 medium supplemented with different lysine concentrations (2, 4, 8, 16, 32, 64, 128, 256 μM). (C) The population density at the saturation phase achieved by the Δ*argH* strain under

different arginine concentrations showed a significant positive correlation with arginine concentration. (D) The saturated-phase population density of the Δ*lysA* strain under different lysine concentrations showed a significant positive correlation with lysine concentration. (C, D) A linear regression was fitted to the data (black line; area between dotted lines: ±95% confidence interval). The results of the spearman correlation analysis were shown. The saturated-phase population density was determined from growth curves by the logistic equation fit calculated using the "Growthcurver" package[2] for *R*.

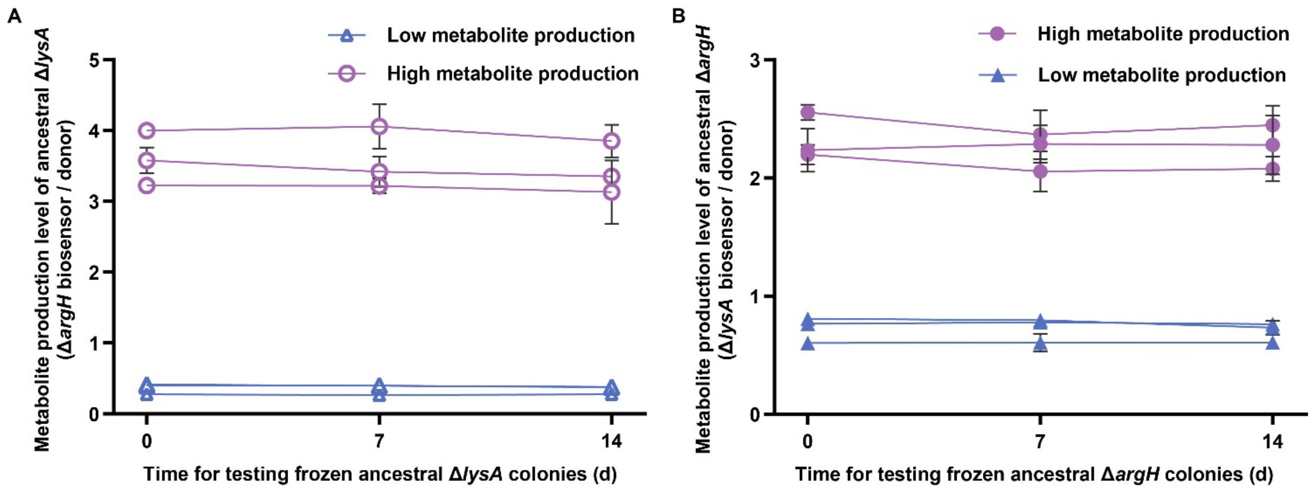

Fig. S6. **Stability of biosensor-based metabolite-production measurements after frozen storage of ancestral Δ*lysA* and Δ*argH* isolates.** Single-colony-derived ancestral isolates were stored as frozen stocks for 0, 7, or 14 days, then revived and assayed for metabolite production under standard biosensor conditions. (A) Metabolite production by ancestral Δ*lysA* isolates after 0, 7, or 14 days of frozen storage. (B) Metabolite production by ancestral Δ*argH* isolates after 0, 7, or 14 days of frozen storage. Purple lines represent high-production isolates and blue lines represent low-production isolates. Each line represents a single isolate. Data points show the mean and s.d. of three technical replicates of each isolate.

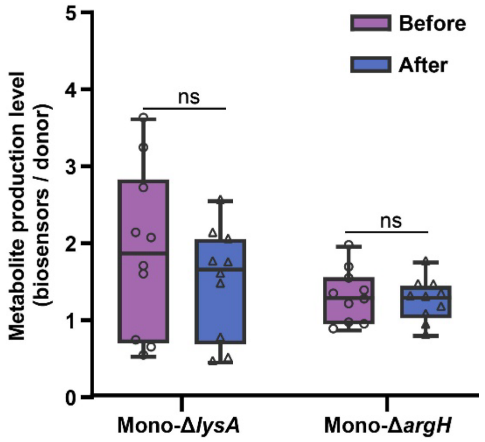

Fig. S7. **Comparison of metabolite production levels between evolved axuotrophs (after) and those of the corresponding ancestors (before) in monoculture.** Evolved axuotrophs were isolated from monoculture treatments at the end of laboratory evolution experiment. Metabolite production level in monoculture before (purple boxplots) and after (blue boxplots) laboratory evolution experiment remained unchanged (paired sample t-tests, Δ*lysA*: p = 0.053, t = 2.231, n = 10; Δ*argH*, p = 0.425, t =0.836, n = 10). In the boxplots, the thick line indicates the median of values, the box the 25th and 75th percentiles, and the whiskers 1.5× the interquartile range from the 25th to the 75th percentile.

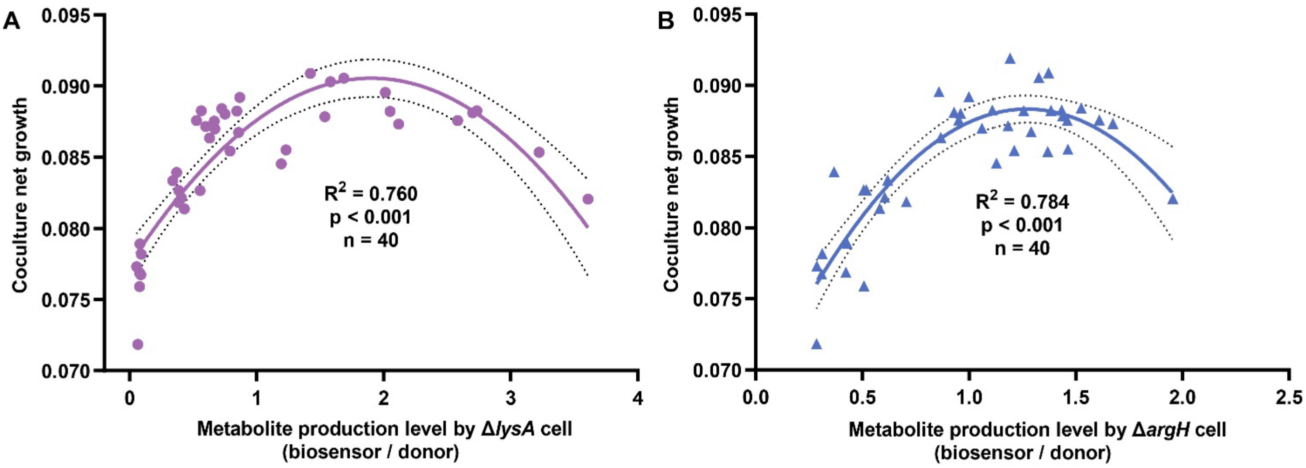

Fig. S8. **Metabolite production by each partner shows a nonlinear relationship with coculture net growth.** Coculture net growth is plotted as a function of metabolite production level by Δ*lysA* (A) or Δ*argH* (B), quantified using the biosensor assay described in Fig. 2. Each point represents an independent coculture measurement across sampling time points (day 0, 3, 15, and 27). Solid curves indicate quadratic regression fits, and the dotted lines denote 95% confidence intervals. In both cases, net growth increases at low to intermediate production levels but plateaus at higher production, consistent with diminishing returns of excessive metabolite supply.

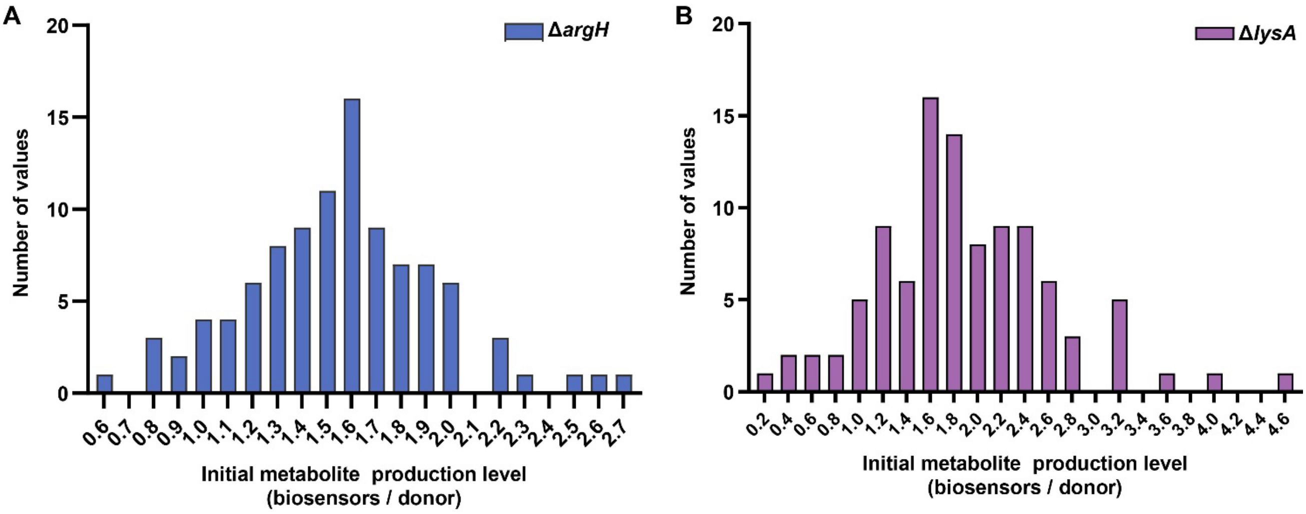

Fig. S9. **Distribution of initial metabolite production levels among 100 independent isolates**. Histograms showing the frequency distribution of initial metabolite production levels for Δ*argH* (A) and Δ*lysA* (B) strains. Production levels are expressed as the ratio of biosensor response to donor cell density (biosensor/donor), providing a normalized estimate of metabolite output per producer. The y-axis indicates the number of isolates within each production interval. Each bar represents one production bin, illustrating the variability in metabolite production across independently isolated strains.

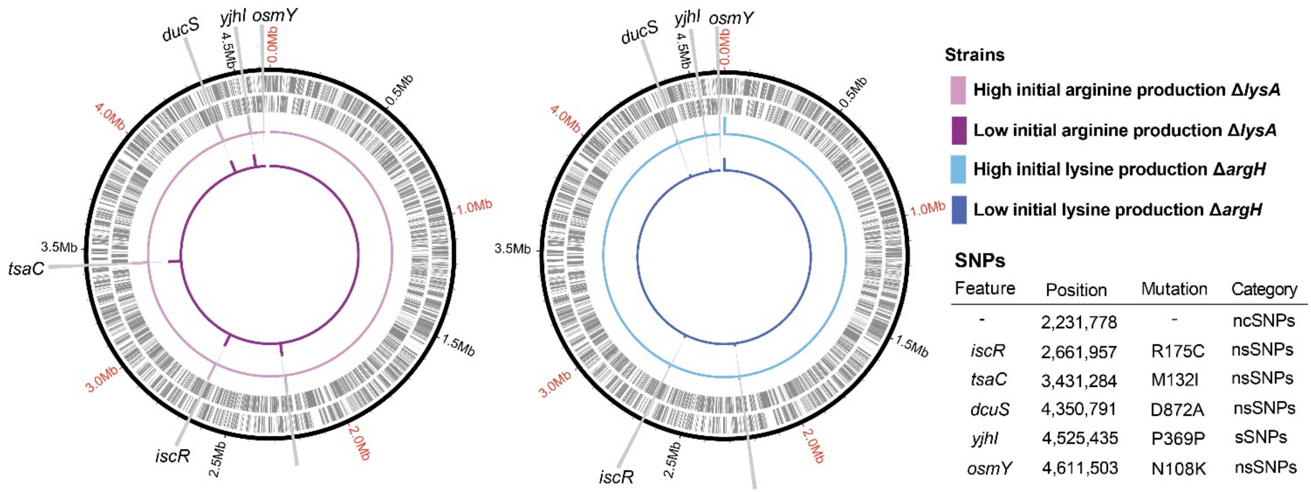

Feature	Position	Mutation	Category
-	2,231,778	-	ncSNPs
iscR	2,661,957	R175C	nsSNPs
tsaC	3,431,284	M132I	nsSNPs
dcuS	4,350,791	D872A	nsSNPs
yjhI	4,525,435	P369P	sSNPs
osmY	4,611,503	N108K	nsSNPs

Fig. S10. **Coding-region variant comparison of of Δ*lysA* and Δ*argH* isolates exhibiting high or low initial metabolite production.** The variant analysis shown here was restricted to annotated coding regions and therefore does not report non-coding or structural variation. Three independent single colonies from each production category were resequenced using Illumina NovaSeq 6000. Concentric rings (outer to inner) indicate the genomic coordinates of the *E. coli* MG1655 reference genome (GenBank: GCF_000005845.2), gene annotations, single-nucleotide polymorphisms (SNPs) detected in high-production isolates, and SNPs detected in low-production isolates. All identified SNPs and their genomic positions are summarized in the table. The same mutations in three sequenced colonies of each strain are shown.

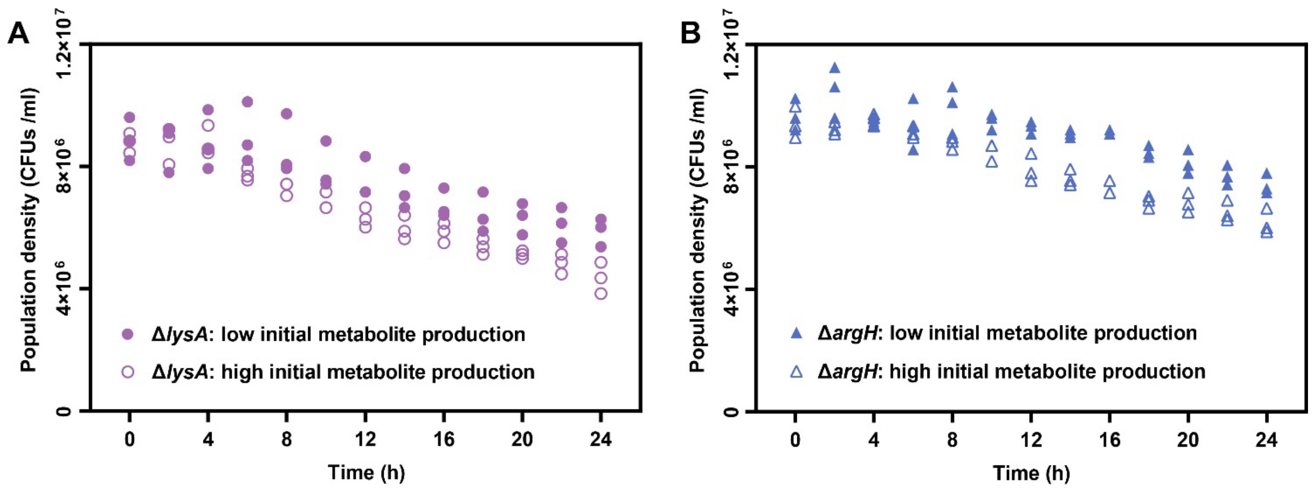

Fig. S11. **Auxotroph populations with low initial metabolite production showed lower population decline.** Overnight cultures of auxotrophs were washed three times using M9 medium and then starved for 4 h to exhaust intracellular stores of amino acids. They were then cultured in M9 medium without supplementation of amino acids for 24 h at 37°C, 220 rpm. Population density (CFU/ml) of Δ*lysA* (A) and Δ*argH* (B) was monitored every 2 h for 24 h. Open symbols represent high-production isolates and filled symbols represent low-production isolates. Purple circles indicate Δ*lysA* and blue triangles indicate Δ*argH*. Points denoted three independent biological replicates.

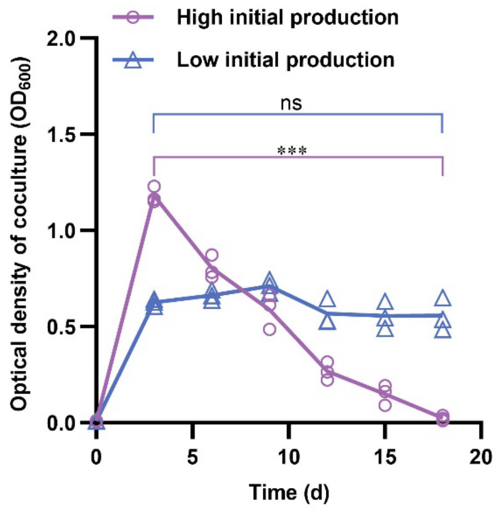

Fig. S12. **Mean population density of coculture quantified as optical density (OD_{600nm}).** Auxotrophic cocultures with high-initial metabolite production (purple line with circles) significantly decreased in optical densities, whereas low-initial metabolite production (blue line with triangles) cocultures remained unchanged (paired sample t test comparing optical densities after 3 days and 18 days of incubation, high: $p < 0.001$, $t = 66.77$, $n = 3$; low: $p = 0.276$, $t = 1.483$, $n = 3$). Points denoted three independent biological replicates.

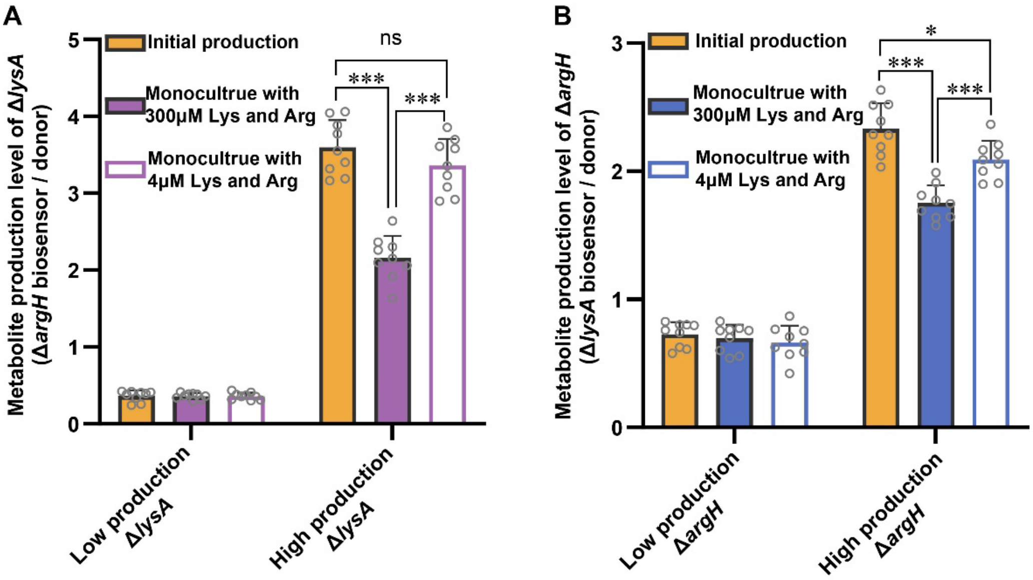

Fig. S13. **High extracellular metabolite availability promoted reduction of metabolite production in initially high-producing auxotrophs**. (A) Metabolite production levels of Δ*lysA* isolates classified as low- or high-initial producers, measured before cultivation (Initial production) and after a 30-day monoculture under metabolite-rich (300 µM lysine and 300 µM arginine) or metabolite-limited (4 µM lysine and 4 µM arginine) conditions. Metabolite production was quantified using the biosensor assay. (B) Corresponding metabolite production levels of low- and high-initial Δ*argH* isolates measured under the same cultivation conditions. Bars represent means ± s.d. Data points showed the three independent biological replicates, each comprising three technical replicates. Statistical significance was assessed using one-way ANOVA followed by Bonferroni post hoc comparisons, where asterisk indicate significant differences among treatments (low production Δ*lysA*: $p = 0.99$, $F = 0.013$; high production Δ*lysA*: $p < 0.001$, $F = 49.06$; low production Δ*argH*: $p = 0.48$, $F = 0.76$; high production Δ*argH*: $p < 0.001$, $F = 28.29$, $df = 26$), and ns denotes no significant difference.

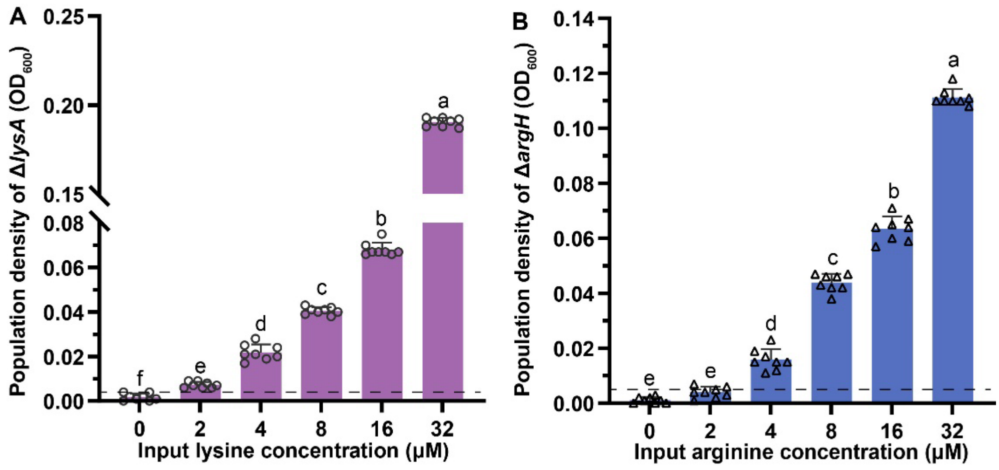

Fig. S14. **The minimum amino acid concentration required for the growth of auxotrophic strains in monoculture.** The minimum amino acid concentration can support a population density (OD_{600}~0.01) of Δ*lysA* (A) and Δ*argH* (B) that doubles relative to the initial density after overnight culture. Points denoted eight independent biological replicates. Mean values (± s.d.) were shown. The dashed line represents the initial density. Different letters represent significant difference among groups (ANOVA followed by a Bonferroni post hoc test: Δ*lysA*, $p < 0.001$, $F = 6960$, $df = 47$; Δ*argH*, $p < 0.001$, $F = 1438$, $df = 47$).

Reference

1 Pande, S. *et al.* Fitness and stability of obligate cross-feeding interactions that emerge upon gene loss in bacteria. *ISME J.* **8**, 953-962 (2014).

2 Sprouffske, K. & Wagner, A. Growthcurver: an R package for obtaining interpretable metrics from microbial growth curves. *BMC Bioinformatics* **17**, 172 (2016).
