# Supplementary material for: Low initial metabolite production enhances stability in syntrophic bacterial consortia
Source: Commun Biol. 2026 May 6;9:938. doi: 10.1038/s42003-026-10187-y (PMC13350956; doi:10.1038/s42003-026-10187-y)
Supplement: Supplementary file 3 — Reporting Summary [file 42003_2026_10187_MOESM3_ESM.pdf]

nature portfolio

Corresponding author(s): Jianxiao Song

Last updated by author(s): Apr 14, 2026

Reporting Summary

Nature Portfolio wishes to improve the reproducibility of the work that we publish. This form provides structure for consistency and transparency in reporting. For further information on Nature Portfolio policies, see our Editorial Policies and the Editorial Policy Checklist.

Statistics

For all statistical analyses, confirm that the following items are present in the figure legend, table legend, main text, or Methods section.

n/a	Confirmed	
☐	☒	The exact sample size (*n*) for each experimental group/condition, given as a discrete number and unit of measurement
☐	☒	A statement on whether measurements were taken from distinct samples or whether the same sample was measured repeatedly
☐	☒	The statistical test(s) used AND whether they are one- or two-sided<br>*Only common tests should be described solely by name; describe more complex techniques in the Methods section.*
☒	☐	A description of all covariates tested
☐	☒	A description of any assumptions or corrections, such as tests of normality and adjustment for multiple comparisons
☐	☒	A full description of the statistical parameters including central tendency (e.g. means) or other basic estimates (e.g. regression coefficient) AND variation (e.g. standard deviation) or associated estimates of uncertainty (e.g. confidence intervals)
☐	☒	For null hypothesis testing, the test statistic (e.g. *F*, *t*, *r*) with confidence intervals, effect sizes, degrees of freedom and *P* value noted<br>*Give P values as exact values whenever suitable.*
☒	☐	For Bayesian analysis, information on the choice of priors and Markov chain Monte Carlo settings
☒	☐	For hierarchical and complex designs, identification of the appropriate level for tests and full reporting of outcomes
☒	☐	Estimates of effect sizes (e.g. Cohen's *d*, Pearson's *r*), indicating how they were calculated

Our web collection on statistics for biologists contains articles on many of the points above.

Software and code

Policy information about availability of computer code

Data collection	1.Gen5 CHS 3.08 was used to collect data about growth curves from the Biotek Synergy Neo2 plate reader.<br>2.NIS-Elements Viewer 5.21 was used to collect data of relative abundance of strains from confocal laser scanning microscope (Nikon AX, Japan).<br>3. QuantStudio Design & Analysis Software v1.5.1 was used to collect qPCR data from Applied Biosystems QuantStudio 5.
Data analysis	1. GraphPad Prism 8 was used for generating most graphs<br>2. All statistical analyses were conducted with SPSS 25.0 (SPSS Inc., Chicago) or R 4.3.2 (https://cran.r-project.org/).<br>3. Microsoft Excel was used to calculate the changes of cell growth OD values, CFU density.<br>4. Adobe Illustrator 2020 was used to assemble figures.

For manuscripts utilizing custom algorithms or software that are central to the research but not yet described in published literature, software must be made available to editors and reviewers. We strongly encourage code deposition in a community repository (e.g. GitHub). See the Nature Portfolio guidelines for submitting code & software for further information.

Data

Policy information about availability of data

All manuscripts must include a data availability statement. This statement should provide the following information, where applicable:

- Accession codes, unique identifiers, or web links for publicly available datasets
- A description of any restrictions on data availability
- For clinical datasets or third party data, please ensure that the statement adheres to our policy

All data used in this manuscript are available on Figshare: https://doi.org/10.6084/m9.figshare.29821316.v1. Additional genomic data generated in this study have been deposited under the BioProject PRJNA1233764 in the NCBI database.

Research involving human participants, their data, or biological material

Policy information about studies with human participants or human data. See also policy information about sex, gender (identity/presentation), and sexual orientation and race, ethnicity and racism.

Reporting on sex and gender	N/A
Reporting on race, ethnicity, or other socially relevant groupings	N/A
Population characteristics	N/A
Recruitment	N/A
Ethics oversight	N/A

Note that full information on the approval of the study protocol must also be provided in the manuscript.

Field-specific reporting

Please select the one below that is the best fit for your research. If you are not sure, read the appropriate sections before making your selection.

☐ Life sciences ☐ Behavioural & social sciences ☒ Ecological, evolutionary & environmental sciences

For a reference copy of the document with all sections, see nature.com/documents/nr-reporting-summary-flat.pdf

Ecological, evolutionary & environmental sciences study design

All studies must disclose on these points even when the disclosure is negative.

Study description	The core experimental design involved the treatment factor being coculture vs. monoculture: Monoculture of ΔlysA in M9 medium + 4 µM Lysine (10 replicates); Monoculture of ΔargH in M9 medium + 4 µM Arginine (10 replicates); Coculture of ΔlysA + ΔargH in M9 medium + 4 µM Lysine + 4 µM Arginine (10 replicates). Subsequent validation experiments explicitly tested the effect of the initial amino acid production level on consortium stability. This involved constructing cocultures with specific combinations: High-production ΔlysA paired with High-production ΔargH (3 biologically independent pairs); Low-production ΔlysA paired with Low-production ΔargH (3 biologically independent pairs). Quantitative data collected throughout the study included optical density (OD600), Colony Forming Units (CFUs), relative fitness calculations, amino acid production levels quantified via a biosensor assay (CFU_biosensor / CFU_donor), strain ratios via fluorescence microscopy, mortality rates, and gene expression data. Statistical analyses included paired t-tests, ANOVA, non-linear regression, and repeated measures ANOVA, with replicate numbers specified for each analysis as detailed in the manuscript methods and figure legends.
Research sample	The parent strain was Escherichia coli MG1655, a standard K-12 laboratory strain obtained from the CCTCC (China Center for Type Culture Collection). Two single-auxotroph strains were constructed from the parent strain via CRISPR-Cas9-mediated gene knockout: a lysine auxotroph (denoted ΔlysA) and an arginine auxotroph (denoted ΔargH). A double-auxotroph "cheater" strain (denoted ΔlysAΔargH) was subsequently constructed by knocking out the argH gene in the ΔlysA background.
Sampling strategy	The main evolution experiment used n = 10 biological replicates per treatment. This sample size was chosen based on established precedents and conventions in microbial experimental evolution and ecology, where 6 to 12 replicates are typically used to balance the detection of population dynamics with experimental feasibility. To assess the natural variation in initial amino acid production levels, 40 independent single colonies each of ΔlysA and ΔargH were randomly selected and measured. For consortia constructed based on specific initial production levels, three biological replicates each of the highest and lowest producing ΔlysA and ΔargH strains were selected from the screened colonies to form pairs. For the introduction of the cheater strain (ΔlysAΔargH) into consortia, n = 3 replicates were used for each initial starting ratio (10:1, 1:1, 1:10) in the low-production or high-production background. Although no formal a priori calculation was conducted in sample size, the chosen sample sizes proved sufficient because: i) They consistently revealed significant and statistically robust differences between experimental groups (e.g., in production levels between

active and inactive cocultures, mortality between high- and low-production strains, and invasion success of the cheater in different backgrounds).ii) Clear and interpretable trends were observed even in groups with smaller replicate numbers (e.g., n=3 for highest and lowest producing pairs). iii) The statistical power was adequate to detect the main effects, as evidenced by the reported p-values (many being $p < 0.01$) and effect sizes (e.g., the high R^2 value for the non-linear regression).

Data collection	Colony Forming Units per milliliter (CFUs/ml) through serial dilution and plating on selective M9 agar plates supplemented with the required amino acid (lysine or arginine). The relative abundance of the mCherry-tagged ΔlysA and GFP-tagged ΔargH strains in coculture was determined using a Nikon AX confocal laser scanning microscope. Images were analyzed using ImageJ software (v1.53s) with a standardized protocol for cell counting. The expression levels of key biosynthetic genes (argH and lysA) were measured via qRT-PCR. To check for coding region differences, resequencing was performed on selected isolates using the Illumina Novaseq 6000 platform. The resulting sequences were compared to the wild-type E. coli MG1655 reference genome (GCF_000005845.2). All data collection followed rigorously defined protocols detailed in the Methods or Supplementary Methods section. All data were collected by Nan Ye and Zhichun Yang.
Timing and spatial scale	The laboratory evolution experiment lasted 27 days, equivalent to approximately 55 generations for the cocultures. The cocultures sampled and transferred to fresh medium every 3 days (a total of 9 transfers).The monocultures transferred daily (1:100 dilution).The 3-day transfer interval for cocultures was chosen to allow sufficient time for the interdependent populations to achieve substantial growth. Daily transfers for monocultures are due to the fact that the strain reaches saturation growth within 24 hours in the M9 medium supplemented with additional amino acids. Stability assay involved a 144-hour period, with a 1:100 dilution at the 72-hour mark. Population density (OD600) and strain ratios were measured every 2 hours. The 72-hour cultivation period before dilution in the stability assay mirrors the transfer period from the evolution experiment, ensuring consistency. The high-frequency (every 2 hours) sampling was essential to capture detailed growth kinetics and population dynamics before and after the perturbation (dilution).
Data exclusions	No data were excluded from the analyses.
Reproducibility	All attempts to repeat the experiment were successful.
Randomization	Transformed E. coli colonies were chosen at random from plates and no data was excluded.
Blinding	The study dose not contain experiments where blinding would be applicable.

Did the study involve field work? ☐ Yes ☒ No

Reporting for specific materials, systems and methods

We require information from authors about some types of materials, experimental systems and methods used in many studies. Here, indicate whether each material, system or method listed is relevant to your study. If you are not sure if a list item applies to your research, read the appropriate section before selecting a response.

Materials & experimental systems

n/a	Involved in the study
☒	☐ Antibodies
☒	☐ Eukaryotic cell lines
☒	☐ Palaeontology and archaeology
☒	☐ Animals and other organisms
☒	☐ Clinical data
☒	☐ Dual use research of concern
☒	☐ Plants

Methods

n/a	Involved in the study
☒	☐ ChIP-seq
☒	☐ Flow cytometry
☒	☐ MRI-based neuroimaging

Plants

Seed stocks	N/A
Novel plant genotypes	N/A
Authentication	N/A
