# Supplementary material for: Low initial metabolite production enhances stability in syntrophic bacterial consortia
Source: Commun Biol. 2026 May 6;9:938. doi: 10.1038/s42003-026-10187-y (PMC13350956; doi:10.1038/s42003-026-10187-y)
Supplement: Supplementary file 1 — Transparent Peer Review file [file 42003_2026_10187_MOESM1_ESM.pdf]

nature portfolio

Peer Review File

Low initial metabolite production enhances stability in syntrophic bacterial consortia

Corresponding Author: Dr Nan Ye

This file contains all reviewer reports in order by version, followed by all author rebuttals in order by version.

Version 0:

Reviewer comments:

Reviewer #1

(Remarks to the Author)
COMMSBIO-25-9586: Low initial amino acid production enhances stability in syntrophic bacterial consortia

This manuscript by Ye et al. presents a comprehensive study examining the auxotrophy-driven microbial interactions in a coculture model system of E. coli. The authors employ an experimental approach that incorporates directed evolution analyses, providing an in-depth examination of microbial adaptation within this community. The manuscript will benefit of additional data. Additionally, several major and minor concerns were identified regarding the justification of the framework and how this is comparable with previous screenings performed for larger amounts of auxotrophs. Overall motivation, methodological clarity, interpretation of findings, and integration of results into the final results need to be further refined, including improvements in visualization and broader comparison with previous findings.

Major comments
1. Introduction. More background on the rationale of the experiment is needed in the introduction, specifically about previous screenings performed for auxotrophic cocultures. Additionally, the antibiotic example needs to be expanded as it was used during the methods.
2. Overall, the manuscript overstates several outcomes that were not measured. Some studies have revealed that auxotrophies can be compensated not only by the exact metabolite for which you are an auxotroph but also by other metabolites in the network. The flexibility of this phenomenon is not addressed in the manuscript, and there is a lack of other multi-omics analyses that can demonstrate its feasibility.
3. There is a lack of state-of-the-art research about why these mutants were selected, making it challenging to identify the novelty in this research.
4. There are interesting tools like biosensors, but they are not adequately explained, and more details about the analysis of these results will enhance the understanding and applicability of this research.

Minor comments
1. Please add the SPSS results to the supplementary information
2. Add the statistical analysis to the overview of Fig. 1

Reviewer #2

(Remarks to the Author)
This manuscript describes cross-feeding between two different E. coli auxotrophs, wherein high amino acid excretion is associated with coculture extinction through serial transfers whereas low amino acid excretion leads to coexistence. The results are interesting, but the interpretation of the results doesn't add up. The narrative is generally written in a clear manner, but the manuscript still needed checking for figure order, results within the methods, and unreferenced figure panels and supplementary methods.

Main concerns:

1. Flawed interpretation of the results. The approach is described as experimental evolution, but then the ‘evolved’ high- and low-production strains are genetically identical (and the ancestor was not sequenced; L317). Phenotypic heterogeneity is used to explain this observation, but heterogeneity doesn’t make sense if the high- and low-production traits are heritable; the authors imply trait inheritance by showing that cultures grown from evolved isolates are phenotypically distinct. I thus question whether (1) mutations were overlooked or (2) the high- and low-production traits are reproducible.

I suspect that mutations were overlooked by the algorithm given that reproducible traits were observed between the two groups (Fig. 4D, Fig 5B+D, Fig 6, Fig S8). Still, these trait examples are all secondary phenotypes. The authors should also show that the primary phenotype of high- and low-amino acid production is reproducible in a figure that includes replicate measurements for each of the isolates that they moved forward with.

I suggest the authors reanalyze the ‘evolved’ isolates. I am not entirely familiar with the approach they used but they only mention looking for SNPs and small indels. What about genome rearrangements? For example, would the algorithm catch DNA inversions that are involved in regulating genes like FimA that lead to biofilm traits (PMID: 2888114; these mutations often popped up in E. coli serial transfers performed by the Lynch and Behringer groups)? The authors should also sequence their ancestor strain rather than assuming it is genetically identical to the reference.

2. I agree with the method that the authors devised to quantify amino acids using ancestral auxotroph strains; sometimes cross-fed metabolites are consumed (including by the producer) as fast as they are produced and thus cannot be detected in supernants. However, I am also concerned about the interpretation that there is contact-dependent cross-feeding (results presented in the methods ~L163). Consider whether the results could also be explained by reacquisition of amino acids by the producer. Separating strains into separate compartments would increase privatization – producer clones would have more opportunity to reacquire amino acids from neighboring cells before they reach the producer. The trends remind me of observations made for ammonium cross-feeding in another coculture system (PMID: 29184014).

I also wonder if nanotubes (L165) might be too controversial to be listed as one of two main modes of amino acid exchange (PMID: 33009406).

3. The similar observations in PMID: 29184014 also made me wonder if the extinction could be explained by evolved competitive bias for some other resource. For example, M9 medium omits several essential trace metals, relying instead on contaminating sources (e.g., from phosphate buffer). These elements are thus potentially limiting. Could strains have evolved to outcompete a partner for trace elements, and in doing so cut itself off from the essential amino acid provided by the out-competed partner? Perhaps some insight could be gained by comparing coculture trends when an evolved strain is cocultures with an evolved partner versus an ancestral partner (e.g., as used for bioassays). However, this again would likely require a mutation(s) that was not observed (see comment 1).

Other concerns:

4. L438 – Maybe the sentence just needs rephrasing, but otherwise I disagree with the notion that excess amino acid excretion would result in product inhibition, thereby limiting amino acid exchange. It sounds contradictory. If high extracellular amino acids are detected, then those amino acids are available to the community. If product inhibition were involved, there should be less amino acids available for detection, not more.

5. I personally find ‘coculture fitness’ to be confusing and unhelpful. Individual fitness is intuitive, but community fitness is not (and I believe it is actively debated). I think the authors can just use net coculture growth as defined in the Figure 3 legend. In this regard, the authors should also provide axis units for Figure 3 (and check other figures); net growth was also omitted from the methods used for the fitting approach (paragraph at L244).

6. L453 – It’s not clear that the authors looked for cheaters in the test cultures, another potential explanation for extinction. Was this because only colonies, and not populations were preserved (see comments on methods below)?

7. I suggest that the amino acid single letter code be used to describe the auxotrophs. It will be difficult for many biologists to unsee L = leucine and A=alanine.

8. Methods need modification to improve clarity:
a. I don’t see that the supplementary methods were referenced.
b. L116 – why is a doubling important? Was the idea to verify some growth but discourage ample growth?
c. L116 - ‘at least double’ the population density could benefit from stating the upper bound. A doubling in Fig S1 isn’t clear from the image itself – consider including the starting density.
d. L118 – ‘rate’ of growth vs ‘amount’ of growth
e. L127 – ‘reactivated’ is unusual. Does this refer to growth of single colonies that were streaked for isolation from frozen stocks?
f. L128 – what is a ‘sample?’ Is it a single colony?
g. L128, 133… – are the 10 colonies used to inoculate 10 cultures? From the description, I can't tell if there were 10 ancestral colonies used to start 10 monocultures and 10 cocultures or 1 ancestral culture started from 10 colonies and then

maybe split into 10 mono- and co-cultures
h. L131 – what was the size of the inoculum? Was it 1% to correspond to the 0.05 OD referenced on L118?
i. L139 – more information is needed to assess how 55 generations was determined. From L121, it appears that monocultures only underwent one doubling (generation) for each transfer due to only being fed 4 uM of amino acid. Does this mean monocultures only underwent 9 generations total?
j. L139 references 9 transfers whereas Fig 1A suggests 100 transfers (100x)
k. L140 – colonies were preserved? Were the colonies grown in monoculture first before preservation? How many colonies were preserved at each time point for each population? Were any populations preserved? Preserving populations would be helpful to revisit to see if 'cheaters' etc, could help explain the observed trends.
l. L142 – I commend the authors for checking to make sure auxotrophs were still auxotrophs by the end of the experiment. But how many colonies were checked? I am trying to get a sense of the frequency at which a low frequency suppressor could be detected. This also makes me wonder again if I understand the monoculture conditions – if only one doubling was possible, then wouldn't full growth indicate a suppressor mutation? Perhaps the authors could also examine what growth trends look like if they introduce a prototroph into the coculture – would it be obvious from growth trends if a suppressor emerged?
m. L163 – this section is results, not methods
n. L271 – I'm not sure that the comparison itself is necessary or helpful, but either way, I felt like I needed more information to assess how these shifting categories from mutualism to amenalism were determined. As described, I am not sure that the approach is appropriate, nor does it seem to align with the visual trends - clearly many cocultures are in decline before day 24. If monocultures were only capable of one doubling, then I don't think the comparison to monocultures is appropriate. Perhaps a more appropriate comparison would be against monocultures that were provided amino acids at levels that do not limit growth.
o. L322 – did I overlook where the methods for determining mortality rates were presented outside of Fig S7 legend? This should be described. As written, it the approach seems to miscommunicate the visual trends in Fig S7. Why were only two data points used when an entire time-course of data is available? The slopes of the death curves look similar.

9. L42, 54, elsewhere? - 'structure' can imply spatial structure whereas I think the authors are trying to describe community composition (a term they use elsewhere).

10. L64 – I advise the introduction deal with topics that the reader will need to interpret the results. Antibiotic responses and riboswitches, for example, are not part of the study. These topics can be left for the discussion.

11. L96 – I don't think the comparative assays used by the authors should be influenced by the fluorescent reporters even if they cause culture growth effects. However, if there are cases where this would be important, I advise the authors trust but verify what has been reported before.

12. L261 and elsewhere – the text frequently mentions results from statistical analyses but omits actual descriptions of the data. In this case, I think it would be informative to describe the range/variation in results across all data points, and not just day 3 and day 27.

13. Standard error is often used but I think standard deviation would be more appropriate for comparing phenotypes from experimental evolution. A metric of variation across lineages (SD) seems more informative than confidence in identifying the mean (SEM); independent lines could take different evolutionary trajectories.

14. Figure panels are often presented out of order.

15. Did I overlook where Figure 1D, E are referenced?

16. Check all figure legends to make sure that error bars and level/nature of replication are described: Fig 5C, Fig S6 (how many colonies were examined for each whisker plot?), others?

17. L360 – rephrasing needed to reflect some similar growth rates shown in the figure

18. L491 – accession number is not available to reviewers through NCBI

Reviewer #3

(Remarks to the Author)
This is a well-executed experimental study that challenges a current assumption in microbial ecology: that higher metabolite production strengthens mutualistic stability. The authors demonstrate, through a combination of experimental evolution, biosensor assays, and invasion analyses, that syntrophic stability emerges from low initial amino acid production, which constrains metabolite leakage and thus limits cheater invasion. The paper provides a novel conceptual insight into how phenotypic heterogeneity shapes mutualistic outcomes. The authors offer a mechanistic explanation rooted in metabolic trade-offs and nonlinear fitness landscapes. While the manuscript is conceptually elegant, methodologically rigorous, and written with clarity, several aspects particularly concerning data interpretation, quantification of heterogeneity, and generalization of findings, could be strengthened before publication. They are as follows:
1. The introduction nicely positions the study within microbial stability theory but should explicitly contrast ecological stability (resilience, persistence) with evolutionary stability (resistance to cheater invasion). I suggest the authors clarify whether the

observed “collapse” represents ecological failure (population decline) or evolutionary instability (trait regression).
2. The idea that “less production = more stability” is novel. However, the generalizability beyond this specific lysine–arginine E. coli system remains uncertain. I recommend a paragraph in the Discussion section exploring whether the principle holds for metabolite exchanges involving public vs. privatized goods (e.g., secreted vitamins, siderophores).
3. The study hinges on the assumption that initial production differences arise from non-genetic phenotypic heterogeneity. While resequencing confirmed no SNP differences, the authors might analyze distribution skewness or bimodality of production levels across colonies to assess whether heterogeneity follows a stochastic or bistable regime.
4. Can the authors report the heritability of production phenotypes across transfers? Do you know if high or low producers retain their identity over several generations?
5. The second-order nonlinear regression (Fig. 3) is elegant, but it should include confidence intervals. Since fitness is derived from growth yields, the authors should clarify whether it represents instantaneous growth rate, yield, or integrated population size. These may differentially reflect resource leakage and cooperation efficiency.
6. The ΔLΔA strain convincingly simulates a non-producer, but its fitness advantage in monoculture (due to removal of biosynthetic cost) could bias competition outcomes. do the authors know the fitness in supplemented medium? Can the authors clarify whether invasion outcomes were measured at steady-state densities or at specific time points.
7. Figures should show replicate data points (not only mean ± SEM) to convey population variance, particularly for Fig. 5 and Fig. 6.
8. The authors can consider adding two key references to their list (which is well-curated and current): consider citing Preussger et al., Current Biology 2020 earlier in the introduction as a framing of reciprocal fitness feedbacks, and D’Souza et al. 2018 Nat Prod Rep. earlier to frame metabolic cross-feeding theory.

Version 1:

Reviewer comments:

Reviewer #1

(Remarks to the Author)
COMMSBIO-25-9586A

The manuscript “Low initial metabolite production enhances stability in syntrophic bacterial consortia” by Ye et al. presents a comprehensive study of auxotrophy-driven microbial interactions in a coculture model of E. coli. The authors employ an experimental approach that incorporates directed evolution analyses, providing an in-depth examination of microbial adaptation within this community. Overall, the authors enhanced their visuals and addressed previously raised questions.
Major comments
1. Authors are trying to tackle a very important issue while studying microbial communities. However, an introduction lacking references is intriguing. The introduction has several statements that are not well sustained without references.
2. Our previous research had shown that communities exchange several kinds of amino acids and other metabolites. To strengthen the manuscript, at least a run of untargeted metabolomics is needed. This can also help explain the different levels of variation across experiments.

Minor
Line 64 Missing a “.”
The figures should be cited in order; please rearrange them. E.g. Fib 1B before 1A.
Line 98- 112 Results. Please add the amino acid concentrations
Fig. 3 name is duplicated

Reviewer #2

(Remarks to the Author)
The manuscript describes outcomes for lysine and arginine E. coli auxotrophs engaged in cross-feeding. Aspects of the manuscript have been improved, including experimentally ruling out some alternative hypotheses suggested by reviewers. The manuscript presents some interesting observations that will be of interest to the field. However, I still found the manuscript to be confusing and there is a concerning lack of transparency.

Major comments.

1. Lack of transparency/ misleading statements. I appreciate that the authors were transparent with the reviewers, but I remain concerned about how this manuscript will be interpreted by readers.

a. In response to reviewer 2 comment 1, the authors acknowledge that they only looked for mutations in coding regions. They state that they explain this limitation in the Discussion (L356-358), but I still think this statement is ambiguous, especially following multiple suggestive statements that there is no genetic basis for the phenotypes. For example: L205 ‘…suggesting that the variability…is not predominantly due to genetic variation’ L206 ‘Despite the lack of coding differences…’ L306 ‘Whole-genome resequencing revealed no consistent differenes in coding regions…’ L308-‘point to regulatory or expression-level heterogeneity as the source of the production variation…’

b. There are many references to 'coding sequences' throughout but never an explicit statement that consideration of all other mutations in coding regions, genome rearrangements, etc were not considered. The reader would have to pay very close attention to catch this. I'm not sure I would believe it if the reviewer response weren't transparent.

c. The authors state that they removed 'phenotypic heterogeneity' (now usually replaced with 'phenotypic variation?'). But L308-'point to regulatory or expression-level heterogeneity as the source of the production variation…' followed by citations of phenotypic heterogeneity/stochastic gene expression, which implies that their conclusions haven't changed despite having identified mutations that aren't being reported in the manuscript. Meanwhile, Fig S6 and S13 show that the high vs low production traits are heritable and consistent through generations. If this is just stochastic gene expression, cultures started from any clonal isolate should give rise to progeny that show similar phenotypic variation.

d. The choice to draw attention to the lack of genome sequence variation by including diagrams in the main figure, while showing expression differences in the supplement, seems to reinforce the possibly incorrect notion that the phenotypes don't have a genetic basis.

e. Suggestion: I think it is fine that the authors have decided that investigating the non-coding mutations is beyond the scope of the current investigation. But, reporting what those mutations are and acknowledging that they could be responsible seems more reasonable and appropriate than risking the reader leave with an entirely different message. I would thus suggest reporting these non-coding mutations. It is odd to only focus on coding regions when whole genome sequences are available. This limitation on data analysis will be unexpected to readers and easily overlooked.

Minor comments.

2. The rebuttal length (25 pages) was counter-productive. I recommend thanking reviewers once upfront and then providing concise responses to each comment. I don't need to know what you intended to convey, etc, in the prior version. Just explain what changes were made and, if necessary, how this improves upon the prior version, or justify why changes aren't being made.

3. Reviewer 3 raised a good point about how generalizable the results are. The authors should acknowledge that expectations of increased reciprocal exchange have a basis in experimental observation. See for example PMID: 39174531 and work by Kost group. This doesn't undermine the novelty of the results in the manuscript.

4. We still don't know why the high-production strains went extinct in coculture. I appreciate that the authors tested several possibilities (e.g., summarized in the abstract) but ultimately the question was left unanswered. I wonder if modeling might help steer experiments towards an answer (e.g., for future work). For example, in the absence of cheaters, are there certain ratios of high and low-producers that would lead to extinction vs coexistence?

5. I like the inclusion of the expression data (why not a main figure). A control that didn't occur to me before would be to also assay expression of amino acid pathways other than the focal amino acids. Is the production specific to the focal amino acids or are these general physiological trends? Do the high production strains also grow faster? Note that high growth rates can correlate with high death rates (PMID: 32500952). I think I will have questions like this as long as not a conclusive explanation for extinction of cocultures with high-production strains.

6. L97 – The previous study by this group came up multiple times in response to reviewer comments, but still seems down-played in the manuscript as a foundational study. I thought this section might benefit from explaining that the first experiments validate observations from the previous study (e.g., lys auxotroph often dominates).

7. Figure panels still mentioned out of order sometimes (e.g., Fig 1A, 2A, others?). This isn't just nitpicking. I would leave this for the copy editor except I had to flip back and forth a lot while reading this manuscript and didn't always find explanations where expected.

8. Monocultures and cocultures were transferred a different number of times. This is now better explained in the methods, but could be better explained in the results and Fig 1A, which only lists 9X transfers, whereas monocultures were transferred 27 times.

9. Fig 1E legend could better explain what is meant by 'Co' and 'Co-<strain name>'

10. L112 '…typically _______ within…' missing word?

11. L133 – The methodology for growing strains in spent supernatants needs more detail. Presumably glucose (and/or nitrogen? 4 uM amino acids?) had to be added to the spent supernatant or was there enough glucose left over too? If nothing is added, then I am confused about what was limiting growth; limiting compounds would still be limiting and inhibitory compounds would still be present. I didn't see more details in the methods.

12. L142 '…standard supernatant measurements did not capture the full extent of exchange…' would benefit from more

explanation

13. L157 ‘Please revise phrasing here and in figure legend to better explain what was actually done. For example, taken literally, metabolite production from 'frozen single-colony isolates' and 'production over a 14-day storage period' doesn't make sense. The statements make it sound like frozen cells are active.

14. Figure 2E onwards are labeled incorrectly (2D missing).

15. Aspects off population decline are still confusing. For example:

a. Reviewer response says that all data was considered but the methods don’t state this explicitly. Referring to initial and final points only in the methods still implies that only two points were used.
b. Fig 4D vs S10 – what is the difference between the values. Are some just negative versions of the other?
c. The supplementary methods state that the values should be negative but Fig 4D shows positive values.

16. L224 – if these are still serial transfers, then I recommend using ‘serial transfers’ to be consistent, rather than introducing new terminology that could imply a different method. Dilution and regrowth implies that fresh media was simply added.

17. Multiple figure panels in the main document and the supplement refer to OD600 values for individual strains in coculture, which shouldn’t be possible. Please use the correct units to indicate how were individual populations assayed in each case. OD600 should not apply when CFUs were counted or when cells were counted microscopically.

18. Figure 6C uses OD/h, but it should be h^-1 for exponential growth. Growth curves could also consider using an exponential y-axis so the reader can visually verify exponential growth.

19. L272 – cultured for one month or serially transferred for one month?

20. There are several cases where similar data sets are presented inconsistently (e.g, in main document vs supplement). Take for example Fig S13 where each panel shows the same kind of data but different conventions are used to show statistical differences. This then requires each convention be explained. Why?

21. L709 – Fig S9 vs S10

22. L720 – lines vs error bars

Reviewer #3

(Remarks to the Author)
The authors have addressed my comments satisfactorily. In particular, the revised manuscript now clarifies the distinction between ecological and evolutionary stability, expands the discussion on generalizability, and provides additional analysis of the distribution of metabolite production among isolates. The revisions improve the clarity of interpretation without changing the core conclusions.

Open Access This Peer Review File is licensed under a Creative Commons Attribution 4.0 International License, which permits use, sharing, adaptation, distribution and reproduction in any medium or format, as long as you give appropriate credit to the original author(s) and the source, provide a link to the Creative Commons license, and indicate if changes were made.
In cases where reviewers are anonymous, credit should be given to 'Anonymous Referee' and the source.
The images or other third party material in this Peer Review File are included in the article’s Creative Commons license, unless indicated otherwise in a credit line to the material. If material is not included in the article’s Creative Commons license and your intended use is not permitted by statutory regulation or exceeds the permitted use, you will need to obtain permission directly from the copyright holder.
To view a copy of this license, visit https://creativecommons.org/licenses/by/4.0/

Point-by-point response to the referees' comments

Note: All author responses are in blue font.

Reviewers' comments:

Reviewer #1 (Remarks to the Author):

COMMSBIO-25-9586: Low initial amino acid production enhances stability in syntrophic bacterial consortia

This manuscript by Ye et al. presents a comprehensive study examining the auxotrophy-driven microbial interactions in a coculture model system of E. coli. The authors employ an experimental approach that incorporates directed evolution analyses, providing an in-depth examination of microbial adaptation within this community. The manuscript will benefit of additional data. Additionally, several major and minor concerns were identified regarding the justification of the framework and how this is comparable with previous screenings performed for larger amounts of auxotrophs. Overall motivation, methodological clarity, interpretation of findings, and integration of results into the final results need to be further refined, including improvements in visualization and broader comparison with previous findings.

Major comments

1. Introduction. More background on the rationale of the experiment is needed in the introduction, specifically about previous screenings performed for auxotrophic cocultures. Additionally, the antibiotic example needs to be expanded as it was used during the methods.

Author response: We thank the reviewer for this constructive comment. We acknowledge that the inclusion of the antibiotic example in the introduction may have caused some confusion. Initially, we intended to highlight how bacterial phenotypic diversity, including persistence cell behavior, might influence their ability to adapt to environments with antibiotics. However, we realize that this example was not directly related to the antibiotic usage described in the methods section. In the methods, antibiotics were specifically used to maintain the fluorescent plasmid tags and were not part of the experimental conditions meant to explore bacterial adaptation to antibiotics. Given the distinct context between the experimental design and the introduction, we have decided to remove the antibiotic example from the introduction to ensure clarity and relevance to the focus of the study. We have carefully considered

the suggestion to provide more background on previous screenings involving auxotrophic cocultures and have rewritten the introduction to provide more context about the challenges associated with stable auxotrophic cocultures. Although auxotrophic strains are commonly used in microbial communities, establishing stable mutually beneficial interactions between two strains is still relatively rare. Typically, stability is achieved when strains are engineered to overproduce specific metabolites. However, recent studies have demonstrated that stability can occur even in the absence of engineered metabolite overproduction. This raises the question: what factors influence the stability of these interactions? Our own prior experiments with lysine- and arginine-auxotrophic strains showed that even under identical experimental conditions, some cocultures will maintain stable growth, while others rapidly become extinct. We hypothesize that these differences may arise from initial phenotypic variation within each strain at the time of community assembly.

2. Overall, the manuscript overstates several outcomes that were not measured. Some studies have revealed that auxotrophies can be compensated not only by the exact metabolite for which you are an auxotroph but also by other metabolites in the network. The flexibility of this phenomenon is not addressed in the manuscript, and there is a lack of other multi-omics analyses that can demonstrate its feasibility.

Author response: We thank the reviewer for raising this important point about metabolic flexibility in auxotrophic systems. We acknowledge that under specific conditions, auxotrophies can be partially compensated by metabolites other than the target missing compound, and such flexibility has been demonstrated in previous studies. In the revised manuscript, we have added experiments to validate whether our strains exhibit metabolic flexibility. As shown in revised Fig. 1B, although Δ*lysA* and Δ*argH* strains cannot grow in the absence of their respective required amino acids, supplementation with the focal amino acid together with other amino acids significantly enhances growth relative to supplementation with the focal amino acid alone. This demonstrates that auxotrophic strains can utilize additional metabolites once primary growth limitation is relieved (lines 98-105 and Supplementary Method in the revised manuscript). Consistent with this observation, we deliberately interpret biosensor readouts as all of potential metabolite production levels, rather than production of a single specific amino acid. The biosensor assay reports the net availability of growth-supporting metabolites accessible to the partner strain,

integrating contributions from the focal amino acid and other metabolites present in the metabolic network. We have clarified this interpretation in the methods section and revised the text and figure legends accordingly (lines 151-157, 442-445, 670-672 in the revised manuscript). Finally, while multi-omics approaches can provide valuable mechanistic insights into metabolic networks, our study is explicitly focused on phenotypic and ecological outcomes of metabolite exchange, rather than on reconstructing the full metabolic repertoire underlying these interactions. The biosensor-based assay, validated by its linear response to metabolite availability (Supplementary Fig. S5), provides a quantitative, system-level readout of metabolite exchange that is sufficient to address our central questions regarding syntrophic stability and evolutionary dynamics. We have therefore chosen a functional, ecology-oriented approach rather than an omics-based descriptive.

3. There is a lack of state-of-the-art research about why these mutants were selected, making it challenging to identify the novelty in this research.

Author response: We agree that the rationale for selecting the Δ*lysA*-Δ*argH* system requires more clarity. In the revised introduction, we now justify our choice of model system based on prior experimental evidence rather than convenience or convention (lines 80-84 in the revised manuscript). We now explain that this *E. coli* lysine-arginine auxotrophic coculture has previously shown repeatably divergent outcomes under identical experimental conditions, with most replicates going extinct and a minority remaining stable. These properties make the system well-suited for addressing our key scientific question, as posited at the end of the now re-written introduction.

4. There are interesting tools like biosensors, but they are not adequately explained, and more details about the analysis of these results will enhance the understanding and applicability of this research.

Author response: We thank the reviewer for this constructive comment. Biosensors have been widely used to infer metabolite production in microbial cross-feeding systems (Bertels et al. 2012, Preussger et al. 2020). In the revised manuscript, we have significantly expanded the description and interpretation of the biosensor detection method to clarify its biological principles and analytical framework.

First, we tested if metabolite levels in the supernatants of monocultures are lower than those in cocultures, where metabolites are exchanged. This was confirmed and is now presented in the results (lines 140-157 in the revised manuscript). We think that other methods such as high-performance liquid chromatography (HPLC) would likely have reduced accuracy for quantifying the target metabolites. In the methods section, we now more clearly define the biosensor used as a growth-based biosensor, where the biomass accumulation of an auxotrophic recipient strain serves as an integrative readout of metabolite availability provided by a cocultured donor. Since the growth of the recipient strain depends entirely on the metabolites released or made available by the partner strain, the biosensor growth value becomes an effective measure of metabolite exchange. We now more clearly emphasize that the aim of this detection system is to reflect the metabolic flexibility of auxotrophic strains (Figure 1B, see also the previous response). Consequently, we interpret the biosensor readings throughout the manuscript as reflecting the net availability of metabolites that support growth, rather than simply the production of a single-target amino acid. Furthermore, we have expanded the description of our data analysis. Specifically, metabolite production is quantified as the ratio of biosensor to donor cell density (CFU(biosensor) / CFU(donor)). This metric controls for the expected positive correlation between donor growth and biosensor growth, and provides an indicator of how many biosensor cells can be supported per donor cell. This method allows for direct comparisons of metabolite production across different donor genotypes at different time points (Revised manuscript lines 140-160, 438-448). Finally, to support the quantitative interpretation of biosensor readings, we present in the results section that, over a wide concentration range, biosensor growth shows a linear positive correlation with external metabolite concentrations (Supplementary Fig. S5). This validates biosensor biomass as a quantitative surrogate for metabolite availability.

Minor comments

1. Please add the SPSS results to the supplementary information

Author response: Thank you for your suggestion. The SPSS statistical analysis results are presented in the results section, and the figure legends. We have also added the SPSS results to the Supplementary Information section. The updated Supplementary Table S2 contains the full set of SPSS results.

2. Add the statistical analysis to the overview of Fig. 1

Author response: Thank you for your suggestion. We have updated the figure legend for Fig 1 to include the appropriate statistical analysis (lines 649, 653, 655, 663, 665 in the revised manuscript). Now, all figures with statistical significance annotations include statistical results in their legends.

Reviewer #2 (Remarks to the Author):

This manuscript describes cross-feeding between two different E. coli auxotrophs, wherein high amino acid excretion is associated with coculture extinction through serial transfers whereas low amino acid excretion leads to coexistence. The results are interesting, but the interpretation of the results doesn't add up. The narrative is generally written in a clear manner, but the manuscript still needed checking for figure order, results within the methods, and unreferenced figure panels and supplementary methods.

Main concerns:

1. Flawed interpretation of the results. The approach is described as experimental evolution, but then the 'evolved' high- and low-production strains are genetically identical (and the ancestor was not sequenced; L317). Phenotypic heterogeneity is used to explain this observation, but heterogeneity doesn't make sense if the high- and low-production traits are heritable; the authors imply trait inheritance by showing that cultures grown from evolved isolates are phenotypically distinct. I thus question whether (1) mutations were overlooked or (2) the high- and low-production traits are reproducible.

I suspect that mutations were overlooked by the algorithm given that reproducible traits were observed between the two groups (Fig. 4D, Fig 5B+D, Fig 6, Fig S8). Still, these trait examples are all secondary phenotypes. The authors should also show that the primary phenotype of high- and low-amino acid production is reproducible in a figure that includes replicate measurements for each of the isolates that they moved forward with.

I suggest the authors reanalyze the 'evolved' isolates. I am not entirely familiar with the approach they used but they only mention looking for SNPs and small indels. What about genome rearrangements? For example, would the algorithm catch DNA inversions that are involved in regulating genes like FimA that lead to biofilm traits

(PMID: 2888114; these mutations often popped up in E. coli serial transfers performed by the Lynch and Behringer groups)? The authors should also sequence their ancestor strain rather than assuming it is genetically identical to the reference.

Author response: We thank the reviewer for these constructive suggestions. We agree that the original wording of the manuscript led to confusion regarding the evolution and divergence of the strains, and the interpretation of trait variation. We have therefore substantially revised the manuscript to clarify these points and to address the reviewer's concerns.

First, we now make clear that the high- and low-production strains analyzed in this study are not "evolved" strains. All isolates used for resequencing and assessing variation in metabolite production (Fig. 4) were obtained as independent single colonies from the frozen glycerol stocks. They were not subjected to coculturing or serial passaging prior to analysis. Thus, these isolates represent parallel genotypes at the same time period as the ancestral strain in the evolution experiment. We have corrected the terminology throughout the manuscript to avoid referring to these isolates as evolved strains (lines 193-197, 463-466 in the revised manuscript). To address the observation that isolates of the same strain showed different metabolite production levels, we resequenced the ancestral high- and low-producing isolates and analyzed coding regions only. No differences were detected in protein-coding sequences. As the reviewer correctly notes, our analysis focused on SNPs and small insertions or deletions and cannot detect structural variants or regulatory rearrangements. We now explicitly acknowledge this limitation in the discussion, and note that future work should include sequencing of the ancestral strain and targeted analyses of structural variation (lines 356-360 in the revised manuscript). In addition, quantitative PCR revealed consistent and significant differences in expression of key biosynthetic genes between high- and low-production isolates (lines 209-212, 306-310 in the revised manuscript). These data support the interpretation that the initial differences in metabolite production are likely due to regulatory variation in non-coding regions. We note, however, that gene regulation in microorganisms is complex and often involves the combined action of multiple regulatory elements. Our conclusion therefore remains limited to the inference that non-coding regulatory variation contributes to the observed differences in initial metabolite production, and the underlying molecular mechanisms were not addressed in the present study. With respect to genetic changes arising during experimental evolution, we agree that

selection is expected to have driven genomic alterations. Ongoing sequencing has identified candidate mutations in several genes. Functional validation and analysis of genetic interactions are in progress. As these experiments require substantial additional work, they are beyond the scope of the current manuscript and will be presented separately.

Second, we have removed the term 'phenotypic heterogeneity' from the manuscript and no longer invoke phenotypic heterogeneity as a mechanistic explanation for community stability. Instead, we now describe the observed differences as intrinsic variation in initial metabolite production among clonal founder colonies. These initial differences are present at the time of community assembly and serve as a source of variation that may bias subsequent ecological outcomes, without inferring stable changes to strain genotypes.

Third, regarding repeatability of primary phenotype production, we have added new data explicitly demonstrating that metabolite production levels are reproducible across time. In Supplementary Fig. S6, we show repeated measurements of metabolite production from frozen stocks over weekly intervals, revealing stable production levels. In Supplementary Fig. S13, we further show that when high- and low-production isolates are maintained in monoculture for one month, low-production isolates retain stable metabolite production across all tested amino-acid supplementation conditions. However, high-production isolates show only a modest reduction in production when external amino-acid concentrations are high. These results demonstrate that the production phenotype is reproducible under monoculture. We now present these data in the revised results section (lines 157-160, 269-278 in the revised manuscript). In monoculture evolution controls, metabolite production remained similar to ancestral levels (Supplementary Fig. S7). By contrast, in short-term coculture assays, when ancestral strains were paired with low-production partners, ancestral metabolite production declined markedly within three days (Fig. 2F, G). This rapid shift indicates that metabolite production is likely environmentally responsive and sensitive to social context (lines 176-184, 214-220 in the revised manuscript).

2. I agree with the method that the authors devised to quantify amino acids using ancestral auxotroph strains; sometimes cross-fed metabolites are consumed (including by the producer) as fast as they are produced and thus cannot be detected in

supernants. However, I am also concerned about the interpretation that there is contact-dependent cross-feeding (results presented in the methods ~L163). Consider whether the results could also be explained by reacquisition of amino acids by the producer. Separating strains into separate compartments would increase privatization – producer clones would have more opportunity to reacquire amino acids from neighboring cells before they reach the producer. The trends remind me of observations made for ammonium cross-feeding in another coculture system (PMID: 29184014).

I also wonder if nanotubes (L165) might be too controversial to be listed as one of two main modes of amino acid exchange (PMID: 33009406).

Author response: We agree that our original wording overstated the evidence for strictly contact-dependent cross-feeding, and have now revised the manuscript to reflect a more cautious and less confirmative interpretation. Our primary intention was to test if amino acid exchange could be fully captured by measurements of extracellular supernatants. A biosensor-based assay was therefore designed to integrate amino acid availability arising from both secretion and immediate local uptake, rather than to distinguish specific transport mechanisms. We agree that the reduced growth observed under physical separation can also be explained by enhanced metabolite re-acquisition by producer cells, rather than requiring obligatory contact-dependent transfer. We now explicitly acknowledge this alternative explanation in the text and avoid interpreting the results as evidence for direct contact-dependent exchange per se (lines 143-147 in the revised manuscript). Additionally, we have revised the relevant section to state that spatial proximity enhances effective cross-feeding, without assigning a specific mechanism (lines 147-153 in the revised manuscript). We also removed language implying that physical contact itself is required for amino acid transfer. Regarding nanotubes, we agree that their role in metabolite exchange remains debated. We have therefore removed mention of nanotubes as a possible explanatory mechanism.

3. The similar observations in PMID: 29184014 also made me wonder if the extinction could be explained by evolved competitive bias for some other resource. For example, M9 medium omits several essential trace metals, relying instead on contaminating sources (e.g., from phosphate buffer). These elements are thus potentially limiting. Could strains have evolved to outcompete a partner for trace

elements, and in doing so cut itself off from the essential amino acid provided by the out-competed partner? Perhaps some insight could be gained by comparing coculture trends when an evolved strain is cocultures with an evolved partner versus an ancestral partner (e.g., as used for bioassays). However, this again would likely require a mutation(s) that was not observed (see comment 1).

Author response: Thank you for your useful comments and suggestions. We agree that competition for trace elements could potentially explain some of the observed trends in our coculture system. We therefore performed an additional experiment to assess whether the observed extinctions could be due to limitations in trace elements in the M9 medium. In this experiment, we cocultured the strains for 72 hours, then centrifuged and filtered the coculture medium to remove cells, followed by a second round of coculturing the supernatant with fresh cells for another 72 hours. Importantly, we observed that the growth curves for the first and second rounds of coculture were similar, suggesting that the trace elements in the M9 medium were sufficient for continued growth, with no significant difference was observed between the two incubation periods (lines 131-135 in the revised manuscript, Supplementary Fig. S3). These results suggest that the observed extinction is unlikely to be caused by the depletion of essential trace elements in the medium and more likely reflects other factors, such as metabolic response. In the context of the suggestion to investigate this further using evolved vs. ancestral cocultures, we found that when the evolved strain reduced metabolite production, the ancestral strain responded by significantly reducing the metabolite supply to its ancestral partner (lines 173-184 in the revised manuscript, Fig. 2D, E, F, G).

Other concerns:

4. L438 – Maybe the sentence just needs rephrasing, but otherwise I disagree with the notion that excess amino acid excretion would result in product inhibition, thereby limiting amino acid exchange. It sounds contradictory. If high extracellular amino acids are detected, then those amino acids are available to the community. If product inhibition were involved, there should be less amino acids available for detection, not more.

Author response: We thank the reviewer for identifying this problem and acknowledge that the original wording was potentially ambiguous. We did not intend to suggest that high extracellular amino acid concentrations directly inhibit amino

acid exchange. Our interpretation is that high intracellular or local extracellular amino acid levels may suppress further biosynthesis via feedback inhibition, thereby reducing continued production rather than limiting the availability of already secreted amino acids. If this is so elevated extracellular amino acids are indeed detectable and accessible to all cells in a microbial consortium. However, elevated extracellular amino acids may weaken the incentive or capacity for sustained production, ultimately destabilizing reciprocal metabolite exchange over time. We have therefore removed this problematic sentence from the revised manuscript.

5. I personally find ‘coculture fitness’ to be confusing and unhelpful. Individual fitness is intuitive, but community fitness is not (and I believe it is actively debated). I think the authors can just use net coculture growth as defined in the Figure 3 legend. In this regard, the authors should also provide axis units for Figure 3 (and check other figures); net growth was also omitted from the methods used for the fitting approach (paragraph at L244).

Author response: To eliminate potentially ambiguity we have removed this term throughout the revised manuscript and now use as a clearer alternative “net coculture growth”. In addition, we have added units to all figure axes. Finally, we have revised the methods section to explicitly describe how net coculture growth was calculated and then used in the fitting of curvilinear functions to our least-squares regression (lines 121-129, 185-189, 427-435, 498-506 in the revised manuscript, Fig. 2I, 3).

6. L453 – It’s not clear that the authors looked for cheaters in the test cultures, another potential explanation for extinction. Was this because only colonies, and not populations were preserved (see comments on methods below)?

Author response: We thank the reviewer for highlighting this point. During the evolution experiment, we did not detect the emergence of completely non-producing auxotrophs among the sampled isolates. As stated in the revised methods (lines 419-422 in the revised manuscript), at each transfer we plated coculture samples on selective M9 agar to separate the two auxotrophic strains and then randomly sampled three single colonies per strain per replicate for downstream analyses. We acknowledge that we did not archive whole-population glycerol stocks during the evolution experiment, and therefore cannot retrospectively assay population-level heterogeneity. However, in the later stages of the evolution of inactive cocultures, we

repeatedly observed that all randomly sampled auxotrophic inactive isolates exhibited significantly reduced metabolite production relative to their ancestor (Fig. 2B, C). Based on these observations, made across independent replicates and time points, we refer to these evolved isolates as ‘very low producers’ rather than ‘cheaters’. To directly assess whether invasion by low- or non-producing phenotypes could explain coculture instability, we performed a further experiment using a lysine-arginine double auxotroph (Δ*lysA*Δ*argH*) that is incapable of producing either of the two metabolites exchanged by our focal *E. coli* strains. This new strain represents a non-metabolite producing genotype and provides a stringent test of cheater invasion (Lines 241-246 in the revised manuscript). The new experiment demonstrated that the non-producing strain readily outcompeted high-production consortia but failed to establish and outcompete low-production consortia (Fig. 6). This confirms that reduced metabolite production can disrupt syntrophic systems in evolution experiments.

7. I suggest that the amino acid single letter code be used to describe the auxotrophs. It will be difficult for many biologists to unsee L = leucine and A=alanine.

Author response: We thank you for highlighting this problem and have now revised the nomenclature throughout the manuscript. Where lysine- and arginine-auxotrophic strains feature in the revised manuscript, these have been designated as their gene deletion genotypes: Δ*lysA* and Δ*argH*. All texts, figures, figure legends, and supplementary materials have also been updated accordingly.

8. Methods need modification to improve clarity:

a. I don’t see that the supplementary methods were referenced.

Author response: We thank the reviewer for highlighting this point. We have now explicitly referenced the Supplementary Methods at all relevant points in the main text (lines 99, 212, 469, 709).

b. L116 – why is a doubling important? Was the idea to verify some growth but discourage ample growth?

Author response: We thank the reviewer for these helpful suggestions. We have now clarified both the rationale and terminology of this assay. The intention of the amino acid titration was not to maximize growth. The intention was to identify a minimal supplementation level that permits detectable growth, while avoiding

substantial biomass accumulation. This approach thereby minimized the effects of amino acid excess in subsequent coculture experiments, where excess amino acids may mask interactions between strains within a coculture. We have revised the methods to clarify that a ~2-fold increase was used as a conservative operational threshold (lines 392-399 in the revised manuscript).

c. L116 - ‘at least double’ the population density could benefit from stating the upper bound. A doubling in Fig S1 isn’t clear from the image itself – consider including the starting density.

Author response: We now more clearly state the initial inoculation density (OD_{600}~0.005) and clarify that 4 µM lysine or arginine yielded an approximately twofold increase in population density after overnight incubation. Higher concentrations resulted in significantly greater biomass accumulation. We also clarify that 4 µM was used as an upper limit for amino acid supplementation in all experiments (lines 392-399 in the revised manuscript). In addition, we have revised Fig. S14 (previous Fig. S1) indicate with a dashed line the starting population density, thereby making the degree of population density change clearer.

d. L118 – ‘rate’ of growth vs ‘amount’ of growth

Author response: We have revised the text to more clearly describe this metric as the amount of growth (line 393 in the revised manuscript).

e. L127 – ‘reactivated’ is unusual. Does this refer to growth of single colonies that were streaked for isolation from frozen stocks?

Author response: Here, “reactivated” refers to streaking frozen glycerol stocks onto agar plates to obtain single colonies. For increased clarity, we replaced “reactivated” with “colonies were isolated from frozen glycerol stocks on lysozyme broth (LB) kanamycin agar” (line 403-406 in the revised manuscript).

f. L128 – what is a ‘sample?’ Is it a single colony?

Author response: Here, a “sample” refers to a single colony. We have now clarified this term in the manuscript (lines 403-406 in the revised manuscript).

g. L128, 133… – are the 10 colonies used to inoculate 10 cultures? From the description, I can't tell if there were 10 ancestral colonies used to start 10 monocultures and 10 cocultures or 1 ancestral culture started from 10 colonies and then maybe split into 10 mono- and co-cultures

Author response: We thank the reviewer for highlighting this point. We now clarify that ten independent ancestral single-colony isolates were used, with each colony being used to initiate one monoculture and one coculture. Thus, the monoculture and coculture derived from a given replicate were founded by the same ancestral colony, while the ten replicates were founded by ten independent ancestral colonies (lines 403-410 in the revised manuscript).

h. L131 – what was the size of the inoculum? Was it 1% to correspond to the 0.05 OD referenced on L118?

Author response: It is correct that the inoculum size was 1% (v/v). Specifically, 50 μL of a diluted culture was transferred into 5 mL of fresh medium to start each coculture and monoculture. We have now clarified this in the methods section (lines 406-407 in the revised manuscript).

i. L139 – more information is needed to assess how 55 generations was determined. From L121, it appears that monocultures only underwent one doubling (generation) for each transfer due to only being fed 4 uM of amino acid. Does this mean monocultures only underwent 9 generations total?

Author response: We agree that the original description failed to provide sufficient detail on how "~55 generations" was determined. In the revised methods, we now explicitly state that generations were computed from population doublings for each growth cycle as: g = ln(Nt/N0)/ln(2), where N_0 and N_t are CFU-based cell densities at the beginning and end of a given cycle, respectively. Total generations were obtained by summing generations across all transfer cycles. Using this CFU-based calculation, cocultures underwent ~55 generations over the 27-day experiment (9 transfers). Importantly, the 4 μM amino acid concentration was chosen as the minimum supplementation to ensure measurable growth under our assay conditions. However, generations were not assumed a priori from this threshold. Instead, generations were derived from the observed N_t/N_0 at each transfer. In monoculture, daily 1:100 dilutions result in approximately 6.6-fold regrowth to saturation,

achieving approximately 170 generations by the end of the experiment. We have also clarified the transfer schedule in the revised methods (monocultures: daily 1:100 dilution; cocultures: every 3 days 1:100 dilution), with the generation estimates reported corresponding to the actual transfer regimes (lines 413-419 in the revised manuscript).

j. L139 references 9 transfers whereas Fig 1A suggests 100 transfers (100x)

Author response: Thank you for highlighting this issue. This typographical error in our figure has now been corrected to reflect 9 transfers (9×).

k. L140 – colonies were preserved? Were the colonies grown in monoculture first before preservation? How many colonies were preserved at each time point for each population? Were any populations preserved? Preserving populations would be helpful to revisit to see if ‘cheaters’ etc, could help explain the observed trends.

Author response: Thank you for raising this point. After obtaining the coculture samples at each transfer, cultures were plated on M9 agar supplemented with either lysine or arginine to separate the two strains, respectively. At each time point, three single colonies were randomly selected for each population of each replicate. For each population at each transfer, we then grew each isolate overnight in supplemented M9 (300 µM of the required amino acid), and cryopreserved the resulting monocultures in 15% glycerol at -80°C for downstream analyses. We have now clarified these procedures in the methods section (lines 419-422 in the revised manuscript).

We did not archive whole-population glycerol stocks during the evolution experiment. However, in the later stages of the experiment we repeatedly observed that all randomly sampled auxotrophic isolates exhibited significantly reduced metabolite production relative to their ancestor. We therefore refer to these evolved strains as ‘very low producers’ rather than ‘non-producers’ or ‘cheaters’ (see previous response). Our invasion experiments using the non-metabolite producing double auxotroph strains were designed to represent an extreme-case invasion scenario. This allowed us to directly test whether invasion by such low-/non-producing strains could account for the observed trends within consortia.

l. L142 – I commend the authors for checking to make sure auxotrophs were still auxotrophs by the end of the experiment. But how many colonies were checked? I am

trying to get a sense of the frequency at which a low frequency suppressor could be detected. This also makes me wonder again if I understand the monoculture conditions – if only one doubling was possible, then wouldn't full growth indicate a suppressor mutation? Perhaps the authors could also examine what growth trends look like if they introduce a prototroph into the coculture – would it be obvious from growth trends if a suppressor emerged?

Author response: We thank the reviewer for these thoughtful questions. At each sampling time point, for each replicate population, three single colonies per strain were randomly isolated and tested. This is now clearly stated in the revised methods (see above response, lines 419-422 in the revised manuscript). While this sampling method may not capture rare suppressors, we note that between days 18 and 27, all randomly sampled isolates across inactive populations exhibited significantly reduced levels of metabolite production relative to their ancestral strain. The repeatable recovery of this phenotype after an extended opportunity for evolutionary change was consistent across independent populations. This suggests that low metabolite-production phenotypes had reached high frequency, or near fixation, rather than representing rare variants that escaped detection. The generation of monocultures was also as described above.

m. L163 – this section is results, not methods

Author response: This content has now been moved to the results section (lines 140-157 in the revised manuscript).

n. L271 – I'm not sure that the comparison itself is necessary or helpful, but either way, I felt like I needed more information to assess how these shifting categories from mutualism to amenalism were determined. As described, I am not sure that the approach is appropriate, nor does it seem to align with the visual trends - clearly many cocultures are in decline before day 24. If monocultures were only capable of one doubling, then I don't think the comparison to monocultures is appropriate. Perhaps a more appropriate comparison would be against monocultures that were provided amino acids at levels that do not limit growth.

Author response: We thank the reviewer for these helpful comments. Upon reflection, we realize that the classification of interactions, as initially proposed using the Mitri & Foster (2013, PMID: 24016192) approach, was not central to the primary

goals of our study. This method identifies interactions based on a comparison of target population densities achieved when the target strain is monocultured or cocultured with other strains under the same conditions. If coculture results in a higher population density of the target strain, it indicates a positive effect (+) from the other strain. As mentioned by the reviewer, this comparison itself does not help much in explaining the subsequent results. Based on this, we have decided to remove this section from the manuscript to streamline the focus on the primary observations related to metabolite production and evolutionary trends. We agree that a more meaningful comparison would involve monocultures that were provided amino acids at non-limiting concentrations, which we will consider in future work to better assess the role of nutrient availability in shaping these dynamics.

o. L322 – did I overlook where the methods for determining mortality rates were presented outside of Fig S7 legend? This should be described. As written, it the approach seems to miscommunicate the visual trends in Fig S7. Why were only two data points used when an entire time-course of data is available? The slopes of the death curves look similar.

Author response: We thank the reviewer for highlighting this omission. In the original version, the method for determining mortality rates was only briefly described in the previous figure S7 legend. In the initial submission, mortality was inferred from the change in viable cell numbers over time in nutrient-free medium. However, this process may include limited cell division in addition to substantial cell death. Therefore, the term 'mortality rate' was not strictly accurate. We now use the term 'population decline', which more appropriately captures the net effect of cell death and any residual cell division. Population decline rates are now defined in the Supplementary Methods as the slope of a linear regression fitted to ln-transformed population density over the full 24-h starvation time-course. This approach incorporates all measured time points rather than relying solely on initial and final population densities, providing a better estimate of population decline under metabolite starvation. However, the conclusion that the high-production strain shows a high population decline rate remains unchanged. The figure legend for Fig. S10 (previous S7) has also been revised accordingly.

9. L42, 54, elsewhere? - ‘structure’ can imply spatial structure whereas I think the authors are trying to describe community composition (a term they use elsewhere).

Author response: We thank the reviewer for pointing out this ambiguity. We have revised for clarity the introduction and now consistently use ‘community composition’ when referring to changes in population makeup, rather than the term ‘structure,’ which could indeed be interpreted as spatial organization (lines 42, 117, 120, 131, 179, 231, 462, 712 in the revised manuscript).

10. L64 – I advise the introduction deal with topics that the reader will need to interpret the results. Antibiotic responses and riboswitches, for example, are not part of the study. These topics can be left for the discussion.

Author response: We thank the reviewer for this constructive suggestion. In response, we have revised the introduction to better align with the study's focus and to ensure it provides the necessary context for interpreting our results. We have removed from the introduction text containing antibiotic responses and riboswitches, as they are not central to the experimental system we present. These topics are now only briefly mentioned in the discussion (lines 310-311 in the revised manuscript). The introduction now focuses on two well-established but contrasting observations: (i) auxotrophy and metabolic dependencies are widespread in microbial systems, yet (ii) stable reciprocal cross-feeding among auxotrophs is comparatively rare in laboratory settings unless metabolite overproduction is engineered. By contrasting engineered overproduction-based consortia with systems in which stable coexistence emerges without additional genetic modifications, we introduce the notion that factors other than the degree to which resources are exchanged may affect system stability.

11. L96 – I don’t think the comparative assays used by the authors should be influenced by the fluorescent reporters even if they cause culture growth effects. However, if there are cases where this would be important, I advise the authors trust but verify what has been reported before.

Author response: Our previous work found that fluorescent reporters did not significantly affect the growth of the strains used in this study. This is now stated in the revised manuscript (lines 378-381).

12. L261 and elsewhere – the text frequently mentions results from statistical analyses but omits actual descriptions of the data. In this case, I think it would be informative to describe the range/variation in results across all data points, and not just day 3 and day 27.

Author response: Thank you for this suggestion. We have revised the results section to describe the full time-course dynamics and the range/variation across replicates, rather than focusing only on day 3 and day 27. Specifically, we now (i) describe the overall trajectories of monocultures across transfers (early rise followed by stability for Δ*lysA*; sustained increase for Δ*argH*; Fig. 1C-D), (ii) report that cocultures typically peak early and then decline while diverging among replicates late in the experiment (Fig. 1E), and (iii) explicitly link this divergence to our classification of active vs inactive cocultures by presenting their distinct density trajectories (Fig. 1F). In addition, as requested, we have added descriptions of Fig. 1G-H showing that cocultures begin with relatively even partner abundances but by day 27 inactive cocultures are typically dominated by Δ*lysA*, whereas active cocultures retain higher relative abundance of Δ*argH* (lines 106-120 in the revised manuscript).

13. Standard error is often used but I think standard deviation would be more appropriate for comparing phenotypes from experimental evolution. A metric of variation across lineages (SD) seems more informative than confidence in identifying the mean (SEM); independent lines could take different evolutionary trajectories.

Author response: We have updated the manuscript to replace standard error of the mean (SEM) with standard deviation (SD) in all relevant figures and text.

14. Figure panels are often presented out of order.

Author response: In the revised manuscript, we have reorganized the figure panels to ensure they are presented in the correct order, with the figure legend and main text now accurately reflecting this revised sequence.

15. Did I overlook where Figure 1D, E are referenced?

Author response: We have now added references to Fig. 1D (Now: Fig. 1G) and Fig. 1E (Now: Fig. 1H) to the revised manuscript (Lines 116-119).

16. Check all figure legends to make sure that error bars and level/nature of replication are described: Fig 5C, Fig S6 (how many colonies were examined for each whisker plot?), others?

Author response: As recommended, we have carefully reviewed all figure legends and have added descriptions of the error bars and level of replication to each figure.

17. L360 – rephrasing needed to reflect some similar growth rates shown in the figure

Author response: We have rewritten this sentence as follows: 'Under nutrient-supplemented conditions, this double auxotroph Δ*lysA*Δ*argH* exhibited higher fitness than the Δ*lysA* single auxotroph and similar fitness to Δ*argH*' (Lines 246-250).

18. L491 – accession number is not available to reviewers through NCBI

Author response: We have now made the sequencing data publicly available. The data can now be accessed directly via NCBI. We apologize for any inconvenience this may have caused during the review process.

Reviewer #3 (Remarks to the Author):

This is a well-executed experimental study that challenges a current assumption in microbial ecology: that higher metabolite production strengthens mutualistic stability. The authors demonstrate, through a combination of experimental evolution, biosensor assays, and invasion analyses, that syntrophic stability emerges from low initial amino acid production, which constrains metabolite leakage and thus limits cheater invasion. The paper provides a novel conceptual insight into how phenotypic heterogeneity shapes mutualistic outcomes. The authors offer a mechanistic explanation rooted in metabolic trade-offs and nonlinear fitness landscapes. While the manuscript is conceptually elegant, methodologically rigorous, and written with clarity, several aspects particularly concerning data interpretation, quantification of heterogeneity, and generalization of findings, could be strengthened before publication. They are as follows:

1. The introduction nicely positions the study within microbial stability theory but should explicitly contrast ecological stability (resilience, persistence) with evolutionary stability (resistance to cheater invasion). I suggest the authors clarify

whether the observed "collapse" represents ecological failure (population decline) or evolutionary instability (trait regression).

Author response: We thank the reviewer for this constructive comment. In the revised introduction, we now explicitly distinguish these two concepts (lines 40-43 in the revised manuscript). In our experiments, collapse is defined as population goes extinct: cocultures exhibit strong population decline over serial transfers, such that a consortium fails to recover growth after several cycles of dilution-regrowth (lines 63-66, 113-115 in the revised manuscript). This coculture extinction outcome is directly captured by our time-series measurements of coculture density across replicates, in which the majority of consortia showed little or no net growth at late time points (inactive cocultures). Our data also indicate that this coculture extinction is accompanied by evolutionary instability in cooperative traits. Specifically, in extinction consortia both partners show a progressive decrease in metabolite production over evolutionary time, ultimately reducing metabolite exchange levels insufficient to support their partner's growth. In other words, the extinction of both bacterial strains within consortia coincides with a cooperative trait regression that prevents cross-feeding.

2. The idea that "less production = more stability" is novel. However, the generalizability beyond this specific lysine–arginine E. coli system remains uncertain. I recommend a paragraph in the Discussion section exploring whether the principle holds for metabolite exchanges involving public vs. privatized goods (e.g., secreted vitamins, siderophores).

Author response: We thank the reviewer for these constructive comments. We found that low metabolite production can enhance stability between the two experimental *E. coli* strains but we stipulate that this conclusion is system-specific. In our study, metabolite exchange primarily operates through proximity-based mechanisms, which may not apply to other types of microbial metabolite exchanges. The extent to which this principle holds in other systems will likely depend on the characteristics of the metabolite exchange and the spatial scale in which interactions between different strains/species occur. For metabolites that rely on contact or short-range exchanges, low production limits the spatial reach of the metabolite, benefiting only neighboring cells. This reduces the selective advantage of 'cheaters' that fail to make available lysine and arginine to other cells. In contrast, for highly diffusible

resources such as siderophores, certain vitamins, or signaling molecules, the spatial coupling between producers and beneficiaries is weaker. In these cases, stable cooperation may require mechanisms beyond the regulation of resource production, such as spatial structure or kin selection. We have expanded the discussion to address these points (lines 339-355 in the revised manuscript).

3. The study hinges on the assumption that initial production differences arise from non-genetic phenotypic heterogeneity. While resequencing confirmed no SNP differences, the authors might analyze distribution skewness or bimodality of production levels across colonies to assess whether heterogeneity follows a stochastic or bistable regime.

Author response: We thank the reviewer for this suggestion. In the revised manuscript, we no longer frame our results as relying on a specific assumption of "phenotypic heterogeneity" in the strict sense. Instead, we describe empirical variation in initial metabolite production levels without assigning it a priori to a particular regulatory mechanism. Whole-genome resequencing confirmed the absence of coding-sequence differences between high- and low-production isolates. Moreover, qPCR revealed significant differences in the expression levels of key biosynthetic genes (*argH* or *lysA*). These data support the interpretation that the initial differences in metabolite production are likely due to regulatory variation in non-coding regions (lines 202-214 in the revised manuscript). We now explicitly acknowledge sequencing limitation in the discussion, and note that future work should include sequencing of the ancestral strain and targeted analyses of structural variation (lines 356-360 in the revised manuscript).

In the revised manuscript, we expanded our analysis of production-level distributions by increasing the number of independently isolated single colonies from 40 to 100 isolates per strain (lines 193-197, 463-466, Fig.4A in the revised manuscript, Supplementary Fig. S9). For both Δ*lysA* and Δ*argH*, the distributions showed no evidence of bimodality. Instead, production levels were continuously distributed, with only mild skewness and moderate kurtosis (Δ*lysA*: skewness = 0.65, kurtosis = 1.39; Δ*argH*: skewness = 0.2, kurtosis = 0.55), consistent with a stochastic distribution of production levels. The histograms of distribution of initial metabolite production level were presented in Supplementary Fig. S9. We have incorporated these new data and analyses into the results section, and clearly state that the observed variation reflects

continuous, non-bimodal differences in production capacity (lines 197-201 in the revised manuscript).

4. Can the authors report the heritability of production phenotypes across transfers? Do you know if high or low producers retain their identity over several generations?

Author response: We thank the reviewer for highlighting this point. We did not estimate heritability in a formal quantitative-genetic sense. However, our serial-transfer experiments allow us to address the stability of production across transfers and whether production changes reflect heritable variation. First, when each auxotroph was propagated in monoculture for ~170 generations, the metabolite production level did not show a consistent shift relative to the ancestor (lines 167-170 in the revised manuscript). This suggests that, under monoculture conditions, there was no strong selection for altered production and the phenotype remained stable across transfers. In contrast, during coculture propagation, production levels changed rapidly and in a repeatable direction in response to the partner strain, consistent with selection acting in the social environment. Notably, in added short-term coculture assays, ancestral strains exhibited a pronounced reduction in metabolite production within three days when paired with derived partners that already displayed strongly reduced metabolite production (Fig. 2F, G). These coculture-dependent shifts indicate that production has a heritable component that can respond to selection, while its expression also depends strongly on the interaction context. Overall, our data support a simple interpretation: metabolite production is stable across transfers in monoculture, but can change across transfers in coculture, implying both heritable and environment-dependent components (lines 162-164, 176-184, 214-220 in the revised manuscript).

5. The second-order nonlinear regression (Fig. 3) is elegant, but it should include confidence intervals. Since fitness is derived from growth yields, the authors should clarify whether it represents instantaneous growth rate, yield, or integrated population size. These may differentially reflect resource leakage and cooperation efficiency.

Author response: We agree that including estimates of variation would improve the interpretability of the second-order nonlinear regression in Fig. 3. Because Fig. 3 is presented as a 3D regression surface, any corresponding estimates of variation would likely be unclear and result in occlusion of both the data points and the fitted

surfaces of the plot. This would likely reduce clarity to readers. However, we now include estimates of variation around best fit surfaces of 2D regression “slice” plots, presented in the Supplementary Information. Here 95% confidence intervals are included (Supplementary Fig. S8).

Fitness was not directly taken as an instantaneous growth rate or a final yield from the original cocultures. Instead, at different time points over the duration of evolution experiment, we isolated single colonies from each coculture and then re-established cocultures, all under standardized conditions. Fitness was quantified using the Malthusian parameter, calculated as: ln $(N_{72h} / N_0) / 72$, where N represented consortium density. Our fitness measure therefore represents a time-averaged net growth over a 72-hour growth window, i.e., an integrated outcome based on population change from the initial to the final density. This measure is distinct from an instantaneous exponential-phase growth rate or a pure endpoint yield metric. We have now revised the methods and results sections to more clearly convey our definition (lines 498-506 and Supplementary Fig. S2, S8 in the revised manuscript).

6. The ΔLΔA strain convincingly simulates a non-producer, but its fitness advantage in monoculture (due to removal of biosynthetic cost) could bias competition outcomes. do the authors know the fitness in supplemented medium? Can the authors clarify whether invasion outcomes were measured at steady-state densities or at specific time points.

Author response: In monoculture with supplemented amino acids, we confirmed that the Δ*lysA*Δ*argH* exhibited higher fitness than the Δ*lysA* single auxotroph and similar fitness to Δ*argH* (Fig 6E). This is consistent with our previous study, which showed that auxotrophic strains can gain fitness advantages by reducing metabolite production during the initial stages of similar long-term coculture experiments (lines 246-252 in the revised manuscript). In the invasion assays using the double auxotroph strain, we measured the invasion dynamics at multiple time points, covering both the exponential growth phase and steady-state densities after 84 hours of coculturing. Periodic measurements were taken every 12 hours to capture both early growth dynamics and steady-state outcomes. These experiments showed that the Δ*lysA*Δ*argH* strain was excluded from low-metabolite-producing consortia, but had the potential to invade and coexist with the other two strains in high-metabolite-producing consortia (Fig. 6G-N). We also found that high initial metabolite production in our coculture

system forms a rich pool of extracellular metabolites. We hypothesized that this may stimulate the emergence of low-production phenotypes and conducted experiments showing that populations of high-production strains are initially more likely to be replaced by cells of low-production strains in a metabolite-rich environment. We also observed that when derived low-producer strains interacted with their ancestral strains, ancestral strains rapidly reduced their metabolite production (Fig. 2F, G). Overall, a nutrient-rich environment created by high-production cross-feeding consortia is more likely to stimulate the emergence of low-production individuals. When one strain reduces metabolite production, its feedback signal quickly stimulates its partner strain to reduce metabolite output, ultimately leading to instability and potential extinction of the mutually beneficial system (lines 173-184, 323-325 in the revised manuscript).

7. Figures should show replicate data points (not only mean ± SEM) to convey population variance, particularly for Fig. 5 and Fig. 6.

Author response: In the revised manuscript, we now present in line and bar plots individual replicate data points in addition to means ± SDs.

8. The authors can consider adding two key references to their list (which is well-curated and current): consider citing Preussger et al., Current Biology 2020 earlier in the introduction as a framing of reciprocal fitness feedbacks, and D'Souza et al. 2018 Nat Prod Rep. earlier to frame metabolic cross-feeding theory.

Author response: We have added both references to the revised introduction. D'Souza et al. (2018) is now used to support the logic underlying the widespread occurrence of auxotrophy and metabolic dependencies. Preussger et al. (2020) is cited in our conceptualization of reciprocal fitness feedbacks in auxotrophic systems. Furthermore, the results of Preussger et al. (2020) contrast with other studies showing a requirement of additional gene editing in order to induce metabolite overproduction to achieve stability in these cross-feeding systems. This suggests that additional influencing stability may have been overlooked. The revised introduction now focuses on two well-established but contrasting observations: (i) auxotrophy and metabolic dependencies are widespread in microbial systems; (ii) stable reciprocal cross-feeding among auxotrophs is relatively rare in laboratory settings unless metabolite overproduction is engineered. By contrasting engineered overproduction-based consortia with systems in which stable coexistence emerges without additional genetic

modifications, we introduce the notion of additional, previously unreported factors helping to promote consortia stability.

References

Bertels, F., Merker, H. & Kost, C. Design and Characterization of Auxotrophy-Based Amino Acid Biosensors. *PLOS ONE* **7**, e41349 (2012).

D'Souza, G. *et al.* Ecology and evolution of metabolic cross-feeding interactions in bacteria. *Natural Product Reports* **35**, 455-488 (2018).

Mitri, S. & Richard Foster, K. The Genotypic View of Social Interactions in Microbial Communities. *Annual Review of Genetics* **47**, 247-273 (2013).

Preussger, D., Giri, S., Muhsal, L. K., Oña, L. & Kost, C. Reciprocal Fitness Feedbacks Promote the Evolution of Mutualistic Cooperation. *Current Biology* **30**, 3580-3590.e3587 (2020).

Point-by-point response to the referees’ comments

Note: All author responses are in blue font.

Reviewers' comments:

Reviewer #1 (Remarks to the Author):

COMMSBIO-25-9586A

The manuscript “Low initial metabolite production enhances stability in syntrophic bacterial consortia” by Ye et al. presents a comprehensive study of auxotrophy-driven microbial interactions in a coculture model of *E. coli*. The authors employ an experimental approach that incorporates directed evolution analyses, providing an in-depth examination of microbial adaptation within this community. Overall, the authors enhanced their visuals and addressed previously raised questions.

Author response: We sincerely appreciate the constructive comments provided by the reviewer, both in the previous rounds and for the key details highlighted in this round. These insightful comments have been invaluable in enhancing the quality of our manuscript.

Major comments

1. Authors are trying to tackle a very important issue while studying microbial communities. However, an introduction lacking references is intriguing. The introduction has several statements that are not well sustained without references.

Author response: We have added several references to support key statements in the Introduction, including citations in lines 61, 68, 77, and 86.

2. Our previous research had shown that communities exchange several kinds of amino acids and other metabolites. To strengthen the manuscript, at least a run of untargeted metabolomics is needed. This can also help explain the different levels of variation across experiments.

Author response: We agree that untargeted metabolomics would provide a broader

view of the compounds exchanged in this system and would also help explain variation among replicates. This analysis is beyond the scope of the present study, but we now acknowledge this limitation explicitly in the Discussion and identify untargeted metabolomics and transcriptomics as important next steps (lines 378-382 in revised manuscript).

Minor

Line 64 Missing a "."

The figures should be cited in order; please rearrange them. E.g. Fib 1B before 1A.

Line 98- 112 Results. Please add the amino acid concentrations

Fig. 3 name is duplicated

Author response: We have made the following corrections: (1) Added the missing period at line 64. (2) Reordered figure citations in the main text to match their numbering (Fig. 1A is now cited before Fig. 1B) to ensure sequential consistency. (3) Added explicit amino acid concentrations (300μM of each amino acid) in the Results section (lines 99-104 in revised manuscript) for clarity. (4) Corrected the duplicate Fig. 3 title to ensure it is unique and accurately describes the data.

Reviewer #2 (Remarks to the Author):

The manuscript describes outcomes for lysine and arginine *E. coli* auxotrophs engaged in cross-feeding. Aspects of the manuscript have been improved, including experimentally ruling out some alternative hypotheses suggested by reviewers. The manuscript presents some interesting observations that will be of interest to the field. However, I still found the manuscript to be confusing and there is a concerning lack of transparency.

Author response: We are deeply grateful for the reviewer's thoughtful and detailed comments. The suggestions offered in both the previous and current rounds have significantly contributed to improving the clarity and overall quality of our manuscript.

Major comments.

1. Lack of transparency/ misleading statements. I appreciate that the authors were transparent with the reviewers, but I remain concerned about how this manuscript will be interpreted by readers.

Author response: We agree that our previous wording could be read as overstating what can be concluded from the sequencing data. In the revised manuscript, we state only that no consistent coding-region differences were detected within the regions analyzed. We no longer use wording that implies the phenotype is not predominantly genetic or that non-genetic explanations have been established. We now make the scope of the sequencing analysis explicit before interpreting the result, and we have tempered all statements that could be read as excluding a genetic basis more broadly (Lines 210-217, 312-316, 320-322, 368-374 in the revised manuscript).

a. In response to reviewer 2 comment 1, the authors acknowledge that they only looked for mutations in coding regions. They state that they explain this limitation in the Discussion (L356-358), but I still think this statement is ambiguous, especially following multiple suggestive statements that there is no genetic basis for the phenotypes. For example: L205 '…suggesting that the variability…is not predominantly due to genetic variation' L206 'Despite the lack of coding differences…' L306 'Whole-genome resequencing revealed no consistent differenes in coding regions…' L308-'point to regulatory or expression-level heterogeneity as the source of the production variation…'

Author response: We agree that our previous wording was too strong. We revised all such statements in the Results and Discussion. The manuscript now states that no consistent coding-region differences were identified within the regions examined, while explicitly noting that non-coding and structural causes remain unresolved (Lines 210-217, 312-316, 320-322, 368-374).

b. There are many references to 'coding sequences' throughout but never an explicit statement that consideration of all other mutations in coding regions, genome

rearrangements, etc were not considered. The reader would have to pay very close attention to catch this. I'm not sure I would believe it if the reviewer response weren't transparent.

Author response: In the revised manuscript (Lines 210-217, 312-316, 320-322, 368-374), we now state explicitly in the Results, Supplementary method, Discussion, and Fig. S10 legend that the current variant analysis was restricted to annotated coding regions. This makes clear that non-coding mutations, genome rearrangements, copy-number changes, and other structural variation were not assessed.

c. The authors state that they removed 'phenotypic heterogeneity' (now usually replaced with 'phenotypic variation?'). But L308-'point to regulatory or expression-level heterogeneity as the source of the production variation…' followed by citations of phenotypic heterogeneity/stochastic gene expression, which implies that their conclusions haven't changed despite having identified mutations that aren't being reported in the manuscript. Meanwhile, Fig S6 and S13 show that the high vs low production traits are heritable and consistent through generations. If this is just stochastic gene expression, cultures started from any clonal isolate should give rise to progeny that show similar phenotypic variation.

Author response: We removed wording that presented regulatory or expression-level heterogeneity as the resolved explanation. The revised manuscript now states that expression differences are associated with the production phenotype, but that the underlying cause remains unresolved (Lines 312-316). We also clarify that the phenotype is reproducible after regrowth from single-colony isolates yet responsive to prolonged environmental treatment, arguing against a purely transient stochastic effect (Lines 317-320).

d. The choice to draw attention to the lack of genome sequence variation by including diagrams in the main figure, while showing expression differences in the supplement, seems to reinforce the possibly incorrect notion that the phenotypes don't have a genetic basis.

Author response: We agree. We moved the coding-region comparison to the Supplementary Information and moved the qPCR result into the main figure so that the presentation no longer places disproportionate emphasis on the absence of coding-region differences.

e. Suggestion: I think it is fine that the authors have decided that investigating the non-coding mutations is beyond the scope of the current investigation. But, reporting what those mutations are and acknowledging that they could be responsible seems more reasonable and appropriate than risking the reader leave with an entirely different message. I would thus suggest reporting these non-coding mutations. It is odd to only focus on coding regions when whole genome sequences are available. This limitation on data analysis will be unexpected to readers and easily overlooked.

Author response: We considered this carefully. However, our current workflow did not provide a reliable analysis of non-coding variants, and we did not want to present an incomplete or potentially misleading list. Instead, we now make this limitation explicit throughout the manuscript and restrict our conclusions accordingly (Lines 210-217, 312-316, 320-322, 368-374).

Minor comments.

2. The rebuttal length (25 pages) was counter-productive. I recommend thanking reviewers once upfront and then providing concise responses to each comment. I don't need to know what you intended to convey, etc, in the prior version. Just explain what changes were made and, if necessary, how this improves upon the prior version, or justify why changes aren't being made.

Author response: Thanks, and we have restructured the rebuttal accordingly.

3. Reviewer 3 raised a good point about how generalizable the results are. The authors should acknowledge that expectations of increased reciprocal exchange have a basis in experimental observation. See for example PMID: 39174531 and work by Kost group. This doesn't undermine the novelty of the results in the manuscript.

Author response: We completely agree that the expectation of increased reciprocal exchange is well-grounded in experimental evidence. The Discussion now explicitly acknowledges prior experimental work showing that established mutualisms can evolve increased reciprocal exchange and stronger metabolic entanglement. We also clarify that our results concern the initial assembly stage of the consortium rather than later coevolution (Lines 301-305 in the revised manuscript).

4. We still don't know why the high-production strains went extinct in coculture. I appreciate that the authors tested several possibilities (e.g., summarized in the abstract) but ultimately the question was left unanswered. I wonder if modeling might help steer experiments towards an answer (e.g., for future work). For example, in the absence of cheaters, are there certain ratios of high and low-producers that would lead to extinction vs coexistence?

Author response: We agree that the extinction of high-production consortia is a multifaceted process. We have revised the Discussion to present this mechanism more cautiously and explicitly as a likely interpretation rather than a definitive conclusion. In the manuscript, we now propose that the reduced stability of high-production consortia most likely arises from a cascading failure of metabolic and ecological constraints. Specifically, sustained high metabolite production likely imposes a physiological burden on producers, while excess metabolite release increases environmental accumulation and shifts exchange from a tightly localized interaction toward a more diffusible public good. This would make the system more vulnerable to exploitation. In parallel, under metabolite-rich conditions, high-producing strains rapidly downregulate production (Supplementary Fig. S13), which reduces partner supply (Fig. 2F, G) and may drive population density below the threshold required to sustain cross-feeding, ultimately leading to extinction. Furthermore, we agree that modeling is the logical next step to decouple these variables. As suggested, we have explicitly incorporated this into the Discussion, proposing future theoretical frameworks to test how the balance of production costs, reciprocal benefits, and initial founder ratios dictate the tipping point between coexistence and extinction (Lines

323-342 in the revised manuscript).

5. I like the inclusion of the expression data (why not a main figure). A control that didn't occur to me before would be to also assay expression of amino acid pathways other than the focal amino acids. Is the production specific to the focal amino acids or are these general physiological trends? Do the high production strains also grow faster? Note that high growth rates can correlate with high death rates (PMID: 32500952). I think I will have questions like this as long as not a conclusive explanation for extinction of cocultures with high-production strains.

Author response: Following your suggestion, we have moved the quantitative PCR data (gene expression of *argH* and *lysA*) from the supplementary material to the main text (now integrated into Fig. 4B). Our explanation for the extinction of high-producing strains in coculture is provided in the previous response. We acknowledge the limitations of the present study with respect to transcriptomic analysis. Expression measurements of key biosynthetic genes alone are not sufficient to distinguish between production in specific metabolic pathways and broader physiological states. We therefore consider these measurements as an important direction for future work, which will be necessary to determine whether the observed phenotypes reflect a specific overinvestment in core exchanged metabolites or a more general physiological trade-off between growth and survival (Lines 378-382 in the revised manuscript).

6. L97 – The previous study by this group came up multiple times in response to reviewer comments, but still seems down-played in the manuscript as a foundational study. I thought this section might benefit from explaining that the first experiments validate observations from the previous study (e.g., lys auxotroph often dominates).

Author response: We agree that our previous work (PMID: 39944238) serves as the foundational basis for the current study. The Results now state explicitly that the divergence we observe here recapitulates the key ecological pattern reported in our previous study, namely the tendency for declining cocultures to become dominated by

Δ*lysA* (Lines 119-124 in the revised manuscript).

7. Figure panels still mentioned out of order sometimes (e.g., Fig 1A, 2A, others?). This isn't just nitpicking. I would leave this for the copy editor except I had to flip back and forth a lot while reading this manuscript and didn't always find explanations where expected.

Author response: In the revised manuscript, all figure panels have been assigned in sequential order.

8. Monocultures and cocultures were transferred a different number of times. This is now better explained in the methods, but could be better explained in the results and Fig 1A, which only lists 9X transfers, whereas monocultures were transferred 27 times.

Author response: The transferred time in monoculture now has shown in Figure 1A and in the Results section (Lines 107-109 in the revised manuscript).

9. Fig 1E legend could better explain what is meant by 'Co' and 'Co-<strain name>'

Author response: The definitions of 'Co', 'Co-Δ*lysA*' and 'Co-Δ*argH*' have been added in Figure 1E legend (Lines 672-673 in the revised manuscript).

10. L112 '…typically _______ within…' missing word?

Author response: The missing word, peaked, has been added (Lines 115 in the revised manuscript).

11. L133 – The methodology for growing strains in spent supernatants needs more detail. Presumably glucose (and/or nitrogen? 4 uM amino acids?) had to be added to the spent supernatant or was there enough glucose left over too? If nothing is added, then I am confused about what was limiting growth; limiting compounds would still be limiting and inhibitory compounds would still be present. I didn't see more details

in the methods.

Author response: We agree that the description of the spent-supernatant assay was too brief and have now added more information to the legend of Fig. S3. In this experiment, we did not supplement the harvested supernatants with additional glucose or amino acids, meaning that the exogenously added substances in the medium are not the limiting factors for growth. We think limiting factor in the coculture growth is the exchanged level of metabolites between the derived strains. As previously established by Shou et al. (PMID: 17267602), the persistence of cross-feeding requires the population density to exceed a specific critical threshold. In our system, initial high metabolite production allows the accumulation of a shared metabolic pool sufficient to push the population beyond this threshold. Conversely, in derived consortia, the reduced level of metabolite exchange fails to support a population density high enough to sustain the reciprocal feedback loop, ultimately leading to growth arrest or extinction (Lines 335-338 in the revised manuscript).

12. L142 '…standard supernatant measurements did not capture the full extent of exchange…' would benefit from more explanation

Author response: We have revised this sentence to clarify why standard supernatant measurements did not capture the full extent of exchange in our system. Specifically, shared metabolites may be consumed or re-acquired in the immediate vicinity of cells before they accumulate in the bulk medium, so cell-free supernatants can underestimate the effective metabolite availability experienced during live coculture (Lines 146-149 in the revised manuscript). This interpretation is consistent with our membrane-separation assay, in which exchange decreased markedly despite free diffusion of small molecules, and with previous work showing that communally valuable cross-fed metabolites can be partially privatized through producer re-acquisition (PMID: 29184014).

13. L157 'Please revise phrasing here and in figure legend to better explain what was actually done. For example, taken literally, metabolite production from 'frozen

single-colony isolates' and 'production over a 14-day storage period' doesn't make sense. The statements make it sound like frozen cells are active.

Author response: We agree that the original phrasing was imprecise and could be read as implying that frozen cells remained metabolically active during storage. This was not our intent. We have revised both the main text and the Figure S6 legend to clarify the experimental workflow: single-colony-derived ancestral isolates were stored as frozen stocks for 0, 7, or 14 days, then revived and assayed for metabolite production using the biosensor assay. We now also clarify that the 14-day interval refers to the duration of frozen storage before revival, rather than metabolite production occurring during storage (Lines 163-168 in the revised manuscript).

14. Figure 2E onwards are labeled incorrectly (2D missing).

Author response: All panels in Figure 2 have been labeled in the order mentioned in the text.

15. Aspects off population decline are still confusing. For example:

a. Reviewer response says that all data was considered but the methods don't state this explicitly. Referring to initial and final points only in the methods still implies that only two points were used.

b. Fig 4D vs S10 – what is the difference between the values. Are some just negative versions of the other?

c. The supplementary methods state that the values should be negative but Fig 4D shows positive values.

Author response: The previous response overstated how the decline values were calculated. In the revised manuscript, we now clarify in the Methods that decline was quantified from the change between the first and last sampled points of the analyzed interval, normalized by elapsed time. Fig 4D is just negative versions of the Fig S10. We also revised Fig. 4D (Now Fig. 4C), Fig. S10 (Now Fig. S11), and the corresponding legends to use a single sign convention throughout. Specifically, because the raw decline values are negative, we plot decline magnitude as a positive

quantity for ease of interpretation. We apologize for the confusion caused by the earlier wording.

16. L224 – if these are still serial transfers, then I recommend using 'serial transfers' to be consistent, rather than introducing new terminology that could imply a different method. Dilution and regrowth implies that fresh media was simply added.

Author response: We have standardized the use of the term 'serial transfer' throughout the text and removed redundant descriptions from the Methods section (Lines 247, 481 in the revised manuscript).

17. Multiple figure panels in the main document and the supplement refer to OD600 values for individual strains in coculture, which shouldn't be possible. Please use the correct units to indicate how were individual populations assayed in each case. OD600 should not apply when CFUs were counted or when cells were counted microscopically.

Author response: We agree that labeling individual strain measurements in coculture as OD_{600} is misleading, as OD_{600} cannot distinguish between different strains. In the revised manuscript, we have corrected this issue. For figure 5, we now show the relative abundance of each strain in coculture (instead of OD_{600} for individual strains), and the figure legend has been updated accordingly.

18. Figure 6C uses OD/h, but it should be h^-1 for exponential growth. Growth curves could also consider using an exponential y-axis so the reader can visually verify exponential growth.

Author response: In the revised manuscript, Figure 6C has used 'h^{-1}' for exponential growth now. Growth curves in Figure 6B have used an exponential y-axis.

19. L272 – cultured for one month or serially transferred for one month?

Author response: We have now clarified in the Results section that the cultures

were serially transferred for one month. The revised text specifies the serial transfer regimen (Lines 284-288 in the revised manuscript).

20. There are several cases where similar data sets are presented inconsistently (e.g, in main document vs supplement). Take for example Fig S13 where each panel shows the same kind of data but different conventions are used to show statistical differences. This then requires each convention be explained. Why?

Author response: In the revised manuscript, we have now used the same convention for indicating statistical significance across all similar data sets.

21. L709 – Fig S9 vs S10

Author response: “Figure S10” (Figure S11 now) is correct, and this sentence has been revised (Lines 725 in the revised manuscript).

22. L720 – lines vs error bars

Author response: Following the updates to Figure 5, we have revised the legend accordingly to state that lines represent mean values.

Reviewer #3 (Remarks to the Author):

The authors have addressed my comments satisfactorily. In particular, the revised manuscript now clarifies the distinction between ecological and evolutionary stability, expands the discussion on generalizability, and provides additional analysis of the distribution of metabolite production among isolates. The revisions improve the clarity of interpretation without changing the core conclusions.

Author response: We would like to express our gratitude to the reviewer for their comprehensive and constructive feedback, which have played a crucial role in refining our manuscript and have greatly strengthened its scientific rigor.
